# Supplementary material for: A Targeted Oligonucleotide Enhancer of SMN2 Exon 7 Splicing Forms Competing Quadruplex and Protein Complexes in Functional Conditions
Source: Cell Rep. 2014 Sep 25;9(1):193–205. doi: 10.1016/j.celrep.2014.08.051 (PMC4536295; doi:10.1016/j.celrep.2014.08.051)
Supplement: Document S1. Supplemental Experimental Procedures, Figures S1–S3, and Tables S1–S3 [file mmc1.pdf]

Cell Reports, Volume *9*

Supplemental Information

A Targeted Oligonucleotide Enhancer of SMN2 Exon 7 Splicing Forms Competing Quadruplex and Protein Complexes in Functional Conditions

Lindsay D. Smith, Rachel L. Dickinson, Christian M. Lucas, Alex Cousins, Alexey A. Malygin, Carika Weldon, Andrew J. Perrett, Andrew R. Bottrill, Mark S. Searle, Glenn A. Burley, and Ian C. Eperon

Supplemental Figure 1

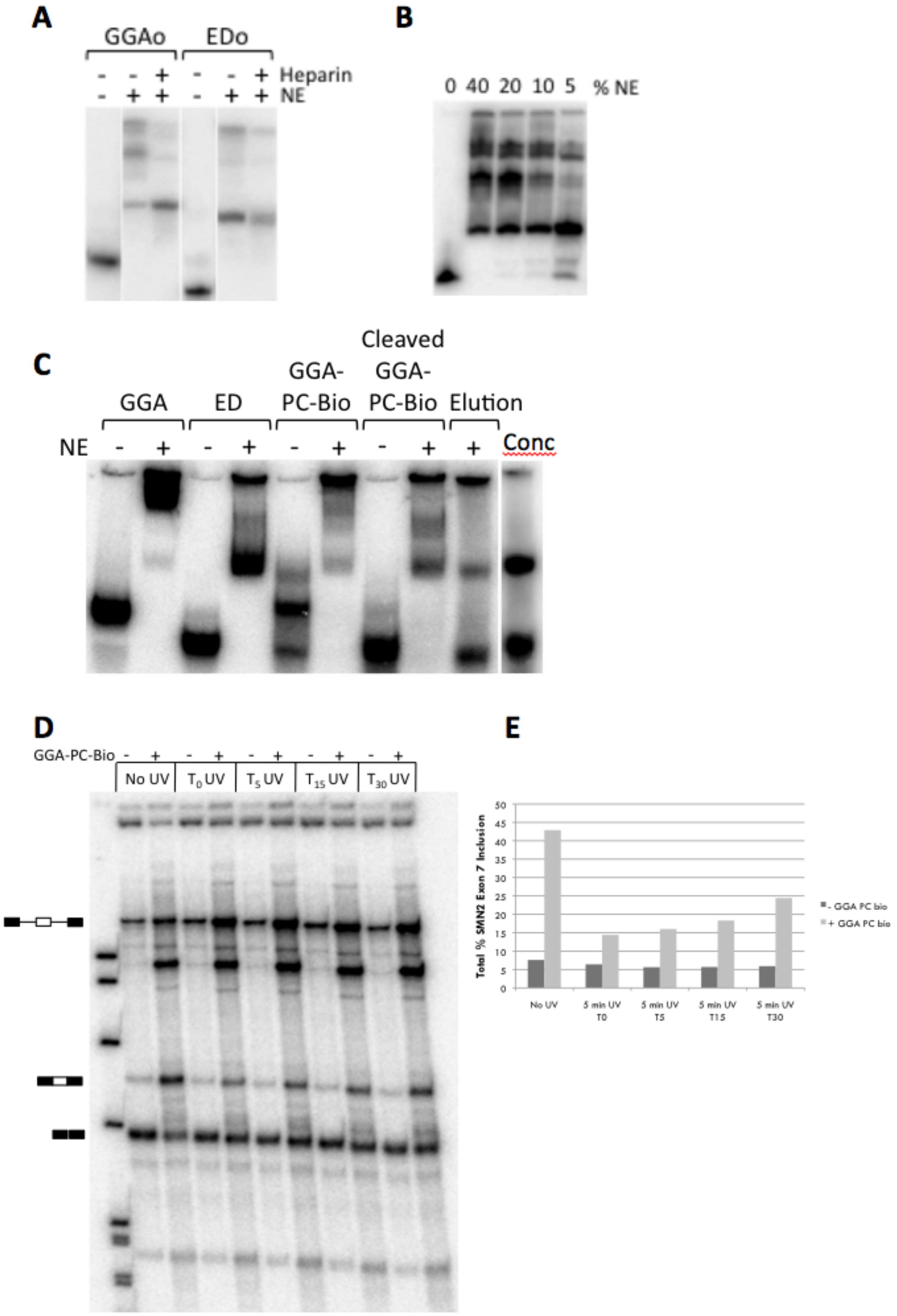

Supplemental Figure 1. Complex formation on photocleavable oligonucleotide. Related to Figure 4.

(A) Formation of heparin-resistant complexes on the ESE domain. Native gel electrophoresis of complexes formed by labelled 2'-O-methyl derivatives of GGA and ED after incubation in nuclear extract (NE) and then heparin, as indicated.

(B) Native gel electrophoresis of complexes formed in various concentrations (%v/v) of nuclear extract.

(C) The ESE domain of GGA forms a complex in nuclear extract equivalent to that formed by ED. A GGA derivative containing a photocleavable linkage between AD and ED (GGA-PC-Bio) was incubated in nuclear extract and irradiated with long wave UV light (for cleavage) prior to native gel electrophoresis. The sample in the right-hand lane was incubated with Neutravidin beads and washed prior to cleavage and analysis of the eluted complex. Conc, eluted material after concentration by ultrafiltration.

(D) Effects of photocleavage of GGA-PC-Bio at different times after initiation of splicing reactions with SMN2 exon 7-containing pre-mRNA. Splicing reactions were incubated for 2 h. Irradiation was done at the times indicated from the start of the reaction.

(E) Level of exon inclusion (inclusion mRNA isoform x 100/[inclusion + exclusion isoforms]) in (D), showing that irradiation did not affect the low level of inclusion in the absence of a TOES oligonucleotide and that its effects on reactions in the presence of GGA-PC-Bio were greater with earlier times of irradiation.

Supplemental Figure 2

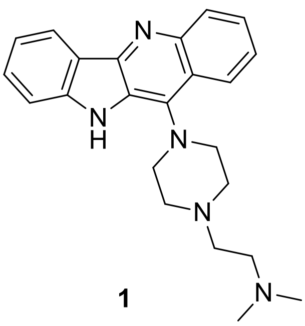

1

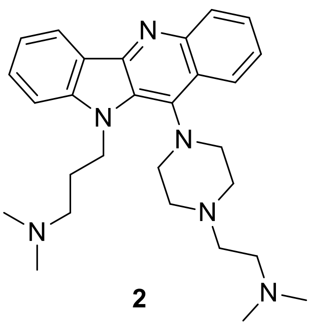

2

Supplemental Figure 2. Structures of quadruplex stabilizers. Related to Figure 6A,B. (1), GSA-0820; (2), GSA-0902. (Boddupally et al., 2012)

Supplemental Figure 3

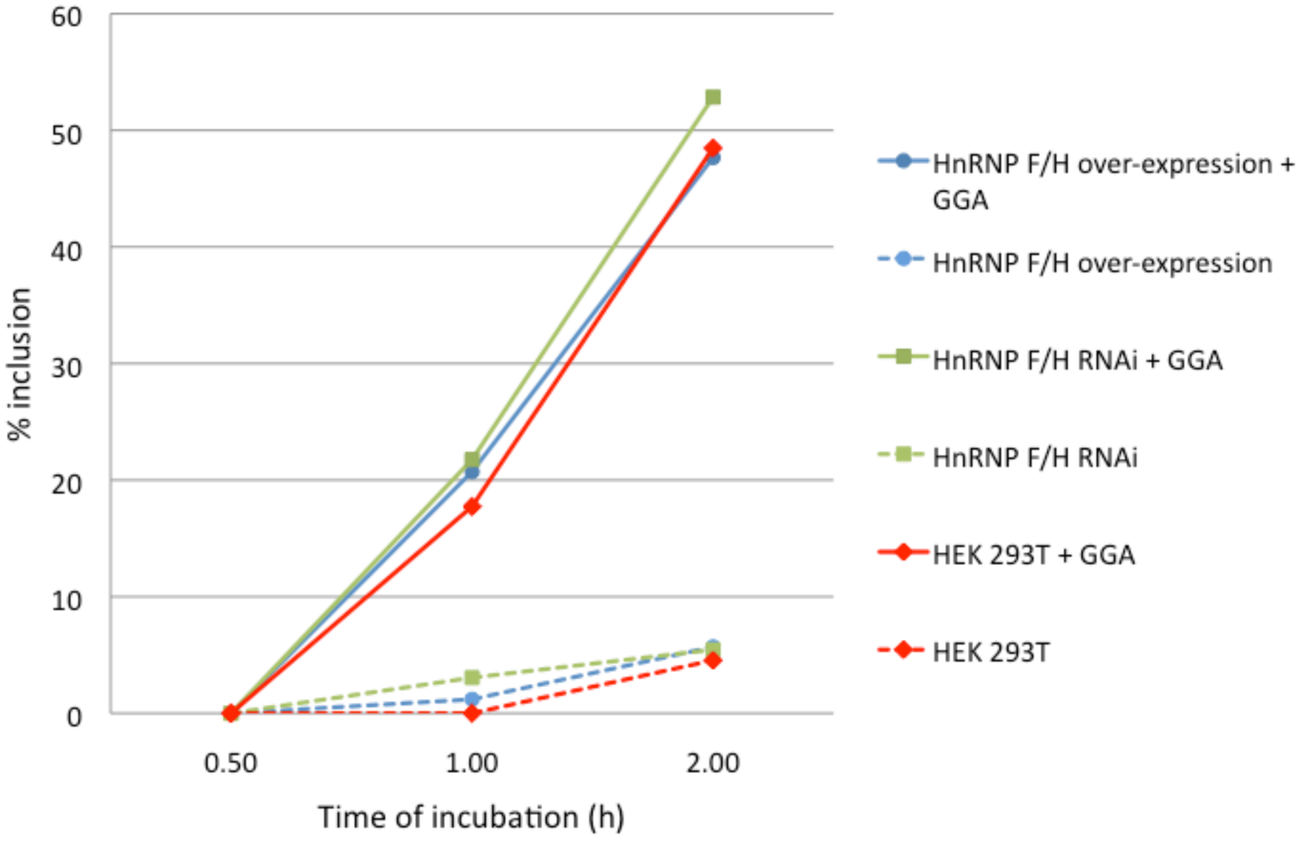

Supplemental Figure 3. Effects of hnRNP F/H depletion and over-expression on activity of the GGA oligonucleotide. Related to Figure 6C. The percentage of inclusion of SMN2 exon 7 was calculated from the phosphor image shown in Figure 6C, where % inclusion is [100*inclusion/(inclusion + exclusion)]. The value changes with time of incubation, probably because the formation of inclusion mRNA (requiring two splicing reactions) is slower than the formation of the skipped or excluded isoform.

Supplemental Table 1.

Rank	Identified Proteins	Accession Number	Mr kDa	Cover-age(%)
1	Probable ATP-dependent RNA helicase DHX36	Q9H2U1	115	53
2	5'-3' exoribonuclease 2	Q9H0D6	109	53
3	Nucleolin	P19338	77	33
4	HnRNP H	G8JLB6 (+1)	51	51
5	HnRNP U-like protein 1	B7Z4B8 (+3)	86	40
6	HnRNP U	Q00839	91	40
7	HnRNP A1	F8VRQ1 (+4)	33	55
8	Double-stranded RNA-binding protein 76	C9JFV5 (+7)	83	40
9	Serine/arginine-rich splicing factor 1	Q07955	28	46
10	Isoform 4 of Cellular nucleic acid-binding protein	P62633-4	20	60
11	CDKN2A-interacting protein	Q9NXV6	61	58
12	HnRNPs A2/B1	P22626 (+1)	37	51
13	Procollagen-lysine,2-oxoglutarate 5-dioxygenase 1	Q02809	84	41
14	HnRNP A3	E7ERJ4 (+2)	34	37
15	RNA-binding protein 45	Q8IUH3 (+1)	54	37
16	Interleukin enhancer-binding factor 2	Q12905	43	28
17	HnRNP H2	P55795	49	47
18	HCG2044799	H3BQZ7 (+1)	85	19
19	Transcriptional activator protein Pur-alpha	Q00577	35	32
20	RNA-binding protein FUS	B4DR70 (+2)	45	27
21	HnRNP F	P52597	46	30
22	5'-3' exoribonuclease 1	Q8IZH2 (+1)	194	6.9
23	Transcriptional activator protein Pur-beta	Q96QR8	33	28
24	U1 small nuclear ribonucleoprotein A	P09012	31	13
25	Serine/arginine-rich splicing factor 2	Q01130 (+1)	25	22
26	TAR DNA-binding protein 43	Q13148 (+1)	45	15
27	HnRNP Q	O60506 (+3)	70	12
28	Serine/arginine-rich splicing factor 5	Q13243	31	20
29	Nuclease-sensitive element-binding protein 1	P67809	36	32
30	Putative ATP-dependent RNA helicase DHX15	F5H6K0 (+1)	90	5.9
31	HnRNP D-like	O14979 (+2)	46	5.2
32	Serine/arginine-rich splicing factor 9	Q13242	26	22
33	Serine/arginine-rich-splicing factor 7	C9JAB2 (+4)	27	13
34	HnRNP R	O43390	71	10
35	Probable ATP-dependent RNA helicase DDX5	E7ETL9 (+1)	62	4.6
36	Serine/arginine-rich splicing factor 6	Q13247 (+1)	40	7.3
37	General transcription factor II-I	B4DH52 (+4)	112	2.2
38	Small nuclear ribonucleoprotein Sm D3	B4DJP7 (+1)	13	16
39	Splicing factor 3B subunit 2	E9PJ04 (+4)	39	9.3
40	TATA-binding protein-associated factor 2N	Q92804 (+1)	62	11
41	Thyroid hormone receptor-associated protein 3	Q9Y2W1	109	2.2

Supplemental Table 1. Ranking of proteins associated with the ED of the GGA oligonucleotide after its release from the AD by photocleavage. Related to Figure 4. Ranking

was done using Top3TIC on Scaffold Viewer, after removal of proteins also identified in a control (-oligonucleotide) preparation. The coverage results refer to the proportion of each protein represented in identified peptides. The probability of identification of each protein was >99.9%.

Supplemental Table 2

	-	H RNAi	F/H RNAi	GFP-H
High Mr	28	39	36	19
GFP-H				26
hnRNP F/H	41	24	22	33
SRSF1	19	23	28	10
CNBP	12	13	15	12

Supplemental Table 2. Relative intensities of proteins crosslinked to 5'-end labelled ED domain in functional HEK293T nuclear extracts. Related to Figure 4D. The values represent the percentage in each band of the total radioactivity in the identified bands.

Supplemental Table 3

**ESE sequence**	**T_m (fold) (°C)**	**T_m (melt) (°C)**
23-mer ESE (2'Ome)	57.5 ±0.1	60.6 ±0.1
23-mer ESE (2'OMe/PS)	66.3 ±0.3	64.9 ±0.2
23-mer ESE (unmodified RNA)	62.2 ±0.1	76.0 ±0.4
15-mer ESE (unmodified RNA)	63.0 ±0.1	75.0 ±0.1

Supplemental Table 3. Apparent T_m values for ESE analogues from CD melting and refolding curves. Related to Figure 5C. Values were recorded at the wavelengths shown in Figure 5C. Transition midpoints were determined based on an apparent two-state model.

Supplemental Experimental Procedures

Protein digestion, analysis by mass spectrometry and data processing. Proteomics was carried out by the University of Leicester Proteomics Facility (PNACL, University of Leicester). Bands of interest were excised from the gel, and in-gel trypsin digestion was carried out upon each (Speicher et al., 2000). Each slice was destained using 200mM ammonium bicarbonate/20% acetonitrile, followed by reduction (10 mM dithiothreitol, Melford Laboratories Ltd., Suffolk, UK), alkylation (100 mM iodoacetamide, Sigma, Dorset, UK) and enzymatic digestion with trypsin (sequencing grade modified porcine trypsin, Promega, Southampton, UK) in 50mM triethylammonium bicarbonate (Sigma) using an automated digest robot (Multiprobe II Plus EX, Perkin Elmer, UK). After overnight digestion, samples were acidified using formic acid (final concentration 0.1%).

LC-MS/MS was carried out using an RSLCnano HPLC system (Dionex, UK) and an LTQ-Orbitrap-Velos mass spectrometer (Thermo Scientific). Samples were loaded at high flow rate onto a reverse-phase trap column (0.3mm i.d. x 1mm), containing 5μm C18 300 Å Acclaim PepMap media (Dionex) maintained at a temperature of 37 °C. The loading buffer was 0.1% formic acid / 0.05% trifluoroacetic acid / 2% acetonitrile. Peptides were eluted from the trap column at a flow rate of 0.3 μl/min and through a reverse-phase capillary column (75μm i.d. x 250mm) containing Symmetry C18 100 Å media (Waters, UK) that was manufactured in-house using a high pressure packing device (Proxeon Biosystems, Denmark). The output from the column was sprayed directly into the nanospray ion source of the LTQ-Orbitrap-Velos mass spectrometer.

The LTQ-Orbitrap-Velos mass spectrometer was set to acquire a 1 microscan FTMS scan event at 60000 resolution over the m/z range 350-1250 Da in positive ion mode. Accurate calibration of the FTMS scan was achieved using a background ion lock mass for polydimethylcyclosiloxane (445.120025 Da). Subsequently up to 10 data dependent HCD MS/MS were triggered from the FTMS scan. The isolation width was 2.0 Da, normalized collision energy 40.0, Activation time 10 ms. Dynamic exclusion was enabled. The .raw data file obtained from each LC-MS/MS acquisition was processed using Proteome Discoverer (version 1.4, Thermo Scientific), searching each file in turn using Mascot (Perkins et al., 1999) (version 2.2.04, Matrix Science Ltd.) against the UniProtKB-Swissprot database. The peptide tolerance was set to 5 ppm and the MS/MS tolerance was set to 0.02 Da. Fixed modifications were set as carbamidomethyl (C) with variable modifications of oxidation (M). A decoy database search was performed. The output from Proteome Discoverer was further processed using Scaffold Q+S (Searle, 2010) (version 3.6.1, Proteome Software). Upon import, the data was searched using X!Tandem (Craig and Beavis, 2004) (The Global Proteome Machine Organization). PeptideProphet (Keller et al., 2002) and ProteinProphet (Nesvizhskii et al., 2003) (Institute for Systems Biology) probability thresholds of

95% were calculated from the decoy searches and Scaffold was used to calculate an improved 95% peptide and protein probability threshold based on the data from the two different search algorithms. Candidate proteins were only accepted if they contained at least 3 peptides >95%. Scaffold was used to calculate the Total Ion Count for the 3 most abundant peptides of each protein for the purposes of protein quantitation.

Structural analysis of oligonucleotides. Unmodified 15-mer and 23-mer samples were from Ribotask (Denmark) and Dharmacon (USA). 2'OMe RNA oligonucleotides were synthesized from DMT-protected β-(cyanoethyl) phosphoramidites (Link Technologies, USA) on 1 μmol CPG supports using an Applied Biosystems 394 nucleic acid synthesizer. RNA oligonucleotides were synthesized from TC-protected phosphoramidites (Link Technologies) (Dellinger et al., 2011). After synthesis, 2'OMe sequences were released by aminolysis (35% aqueous ammonia, 4 h at ambient temperature) and deprotected by heating in 35% aqueous ammonia solution at 55°C for 16h. RNA oligonucleotides were treated in diethylamine/acetonitrile and deprotected with ethylene diamine/toluene prior to elution with triethylammonium acetate. All oligonucleotides were purified by electrophoresis on 10% denaturing gels. Phosphorothioate oligonucleotides were prepared as described (Krotz et al., 2004; Ravikumar et al., 2006). All products were desalted using Illustra NAP™ 25 columns, lyophilised and dissolved in double-distilled H_2O.

For structural analysis, samples were reconstituted in KCl buffer (100mM KCl, 10mM K_2HPO_4/KH_2PO_4, pH 7) made with filtered and degassed water. Prior to use samples were heated to 95°C for 10 minutes and then kept at room temperature for at least an hour to allow secondary structure to form. Unmodified RNA sample concentration was estimated using the nearest neighbour method. CD, UV and thermal denaturation spectra were recorded using a PiStar 180 Spectrophotometer (Applied Photophysics, UK) with strand concentrations between 1-10 μM. Temperature ramping used a rate of 3 min/0.5°C steps and a tolerance of 0.2° C. Data was fitted in OriginPro 8.6 (OriginLab). For NMR, samples were diluted with 10% D_2O to 0.3 to 0.8 mM. Spectra were recorded using a Bruker Avance 600MHz and Bruker Avance III 800MHz with Cryoprobe. The pulse program used was a 1D sequence with water suppression and excitation sculpting with gradients (Hwang and Shaka, 1995). Data was processed in Bruker Topspin 3.1. Mass spectrometry was done in negative ion mode using a Synapt High Definition Mass Spectrometer with Quadrupole TOF mass analyzer (Waters, UK). Samples used in mass spectrometry were buffer-exchanged into 150 mM NH_4OAc, either using a polyacrylamide desalting column (Thermo Scientific) followed by spin concentration to 20-40 μM (23-mer), or dialysis (Slide-a-Lyzer G2, Thermo Scientific, 2K MWCO) to 31 μM (15-mer). Samples were mixed with 10% MeOH immediately before analysis. An injection rate of 4-10 $uL.min^{-1}$ was used and spectra recorded with capillary voltage 1.5-2.5 kV. Data was processed with MassLynx V4.1 (Waters) with smoothing and baseline subtraction.

References

Boddupally, P.V., Hahn, S., Beman, C., De, B., Brooks, T.A., Gokhale, V., and Hurley, L.H. (2012). Anticancer activity and cellular repression of c-MYC by the G-quadruplex-stabilizing 11-piperazinylquindoline is not dependent on direct targeting of the G-quadruplex in the c-MYC promoter. J Med Chem *55*, 6076-6086.

Craig, R., and Beavis, R.C. (2004). TANDEM: matching proteins with tandem mass spectra. Bioinformatics *20*, 1466-1467.

Dellinger, D.J., Timar, Z., Myerson, J., Sierzchala, A.B., Turner, J., Ferreira, F., Kupihar, Z., Dellinger, G., Hill, K.W., Powell, J.A., *et al.* (2011). Streamlined process for the chemical synthesis of RNA using 2'-O-thionocarbamate-protected nucleoside phosphoramidites in the solid phase. J. Am. Chem. Soc. *133*, 11540-11556.

Hwang, T.L., and Shaka, A.J. (1995). Water Suppression That Works - Excitation Sculpting Using Arbitrary Wave-Forms and Pulsed-Field Gradients. Journal of Magnetic Resonance Series A *112*, 275-279.

Keller, A., Nesvizhskii, A.I., Kolker, E., and Aebersold, R. (2002). Empirical statistical model to estimate the accuracy of peptide identifications made by MS/MS and database search. Anal Chem *74*, 5383-5392.

Krotz, A.H., Gorman, D., Mataruse, P., Foster, C., Godbout, J.D., Coffin, C.C., and Scozzari, A.N. (2004). Phosphorothioate oligonucleotides with low phosphate diester content: Greater than 99.9% sulfurization efficiency with "aged" solutions of phenylacetyl disulfide (PADS). Org. Proc. Res. Dev. *8*, 852-858.

Nesvizhskii, A.I., Keller, A., Kolker, E., and Aebersold, R. (2003). A statistical model for identifying proteins by tandem mass spectrometry. Anal Chem *75*, 4646-4658.

Perkins, D.N., Pappin, D.J., Creasy, D.M., and Cottrell, J.S. (1999). Probability-based protein identification by searching sequence databases using mass spectrometry data. Electrophoresis *20*, 3551-3567.

Ravikumar, V.T., Andrade, M., Carty, R.L., Dan, A., and Barone, S. (2006). Development of siRNA for therapeutics: efficient synthesis of phosphorothioate RNA utilizing phenylacetyl disulfide (PADS). Bioorg. Med. Chem. Lett. *16*, 2513-2517.

Searle, B.C. (2010). Scaffold: a bioinformatic tool for validating MS/MS-based proteomic studies. Proteomics *10*, 1265-1269.

Speicher, K.D., Kolbas, O., Harper, S., and Speicher, D.W. (2000). Systematic analysis of peptide recoveries from in-gel digestions for protein identifications in proteome studies. J Biomol Tech *11*, 74-86.
