# Supplementary material for: A Targeted Oligonucleotide Enhancer of SMN2 Exon 7 Splicing Forms Competing Quadruplex and Protein Complexes in Functional Conditions
Source: Cell Rep. 2014 Sep 25;9(1):193–205. doi: 10.1016/j.celrep.2014.08.051 (PMC4536295; doi:10.1016/j.celrep.2014.08.051)
Supplement: Document S2. Article plus Supplemental Information [file mmc2.pdf]

Cell Reports

Article

A Targeted Oligonucleotide Enhancer of SMN2 Exon 7 Splicing Forms Competing Quadruplex and Protein Complexes in Functional Conditions

Graphical Abstract

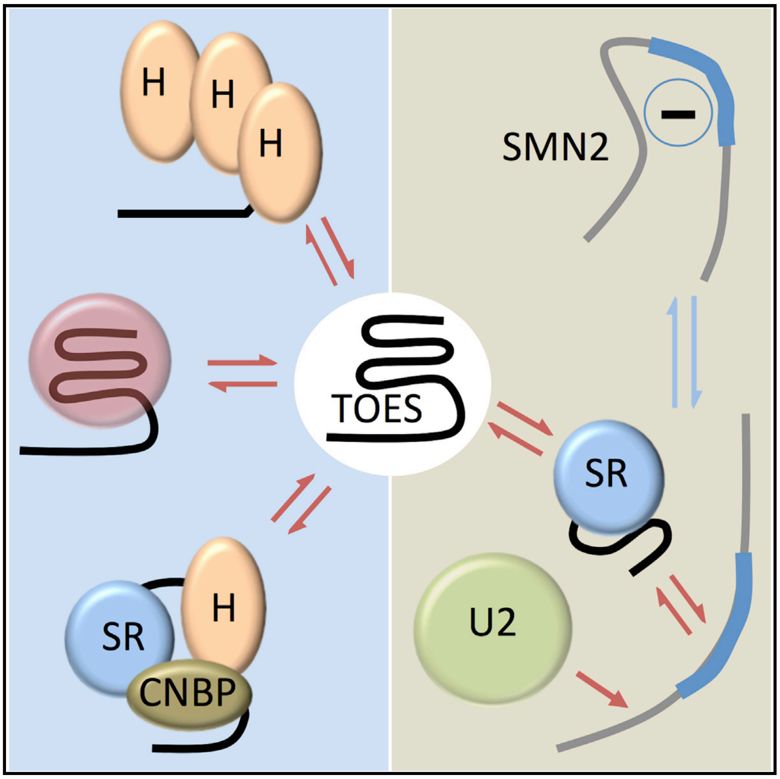

Highlights

The ESE domain of a TOES oligo reduces intron 7-mediated repression of SMN2 exon 7

The ESE stimulates U2 snRNP recruitment when the oligo has annealed

The ESE forms a quadruplex and several discrete nonfunctional protein complexes

Splicing activation may require rapid exchange of proteins or ESE-protein complexes

Authors

Lindsay D. Smith, Rachel L. Dickinson, ..., Glenn A. Burley, Ian C. Eperon

Correspondence

glenn.burley@strath.ac.uk (G.A.B.), eci@leicester.ac.uk (I.C.E.)

In Brief

The use of oligonucleotides to activate selected exons is hampered by a poor understanding of the mechanisms of splicing enhancers. In this study, Smith et al. show that a bifunctional oligonucleotide enhancer of splicing of SMN2 exon 7 stimulates recruitment of U2 snRNP to the upstream intron. Surprisingly, the GGA-rich enhancer forms a quadruplex and complexes enriched in hnRNP H and other proteins. Nonetheless, these complexes do not prevent enhancer activity, suggesting that SR proteins function here via dynamic exchange.

Smith et al., 2014, Cell Reports 9, 193–205
October 9, 2014 ©2014 The Authors
http://dx.doi.org/10.1016/j.celrep.2014.08.051

Cell Reports
Article

OPEN ACCESS
CellPress

A Targeted Oligonucleotide Enhancer of SMN2 Exon 7 Splicing Forms Competing Quadruplex and Protein Complexes in Functional Conditions

Lindsay D. Smith,[1,7] Rachel L. Dickinson,[1,2,7] Christian M. Lucas,[1] Alex Cousins,[3] Alexey A. Malygin,[4] Carika Weldon,[1] Andrew J. Perrett,[2] Andrew R. Bottrill,[5] Mark S. Searle,[3] Glenn A. Burley,[6,*] and Ian C. Eperon[1,*]
[1]Department of Biochemistry, University of Leicester, Leicester LE1 9HN, UK
[2]Department of Chemistry, University of Leicester, Leicester LE1 7RH, UK
[3]School of Chemistry, Centre for Biomolecular Sciences, University of Nottingham, Nottingham NG7 2RD, UK
[4]Institute of Chemical Biology and Fundamental Medicine, Siberian Branch of the Russian Academy of Sciences, Novosibirsk 630090, Russia
[5]Protein and Nucleic Acid Chemistry Laboratory, University of Leicester, Leicester LE1 9HN, UK
[6]Department of Pure & Applied Chemistry, University of Strathclyde, Glasgow G1 1XL, UK
[7]Co-first author
*Correspondence: glenn.burley@strath.ac.uk (G.A.B.), eci@leicester.ac.uk (I.C.E.)
http://dx.doi.org/10.1016/j.celrep.2014.08.051
This is an open access article under the CC BY license (http://creativecommons.org/licenses/by/3.0/).

SUMMARY

The use of oligonucleotides to activate the splicing of selected exons is limited by a poor understanding of the mechanisms affected. A targeted bifunctional oligonucleotide enhancer of splicing (TOES) anneals to SMN2 exon 7 and carries an exonic splicing enhancer (ESE) sequence. We show that it stimulates splicing specifically of intron 6 in the presence of repressing sequences in intron 7. Complementarity to the 5′ end of exon 7 increases U2AF65 binding, but the ESE sequence is required for efficient recruitment of U2 snRNP. The ESE forms at least three coexisting discrete states: a quadruplex, a complex containing only hnRNP F/H, and a complex enriched in the activator SRSF1. Neither hnRNP H nor quadruplex formation contributes to ESE activity. The results suggest that splicing limited by weak signals can be rescued by rapid exchange of TOES oligonucleotides in various complexes and raise the possibility that SR proteins associate transiently with ESEs.

INTRODUCTION

Pre-mRNA splicing has the potential to be a target of considerable importance for therapeutic intervention. Most human protein-coding genes express two or more spliced isoforms of mRNA at significant levels, conferring additional diversity and flexibility to the informational capability of a limited number of genes (Djebali et al., 2012; Pan et al., 2008; Wang et al., 2008). Moreover, recent reports suggest that splicing might be stochastic, i.e., that the use of a particular exon or splice site is a matter of probability and that many minor alternative events might arise as stochastic noise (Djebali et al., 2012; Melamud and Moult, 2009). If splicing patterns are not fixed, it may be feasible to redirect almost any splicing pattern for therapeutic purposes.

One of the most successful techniques for redirecting the splicing patterns of specific genes is to use oligonucleotides complementary to splicing signals or auxiliary motifs in the pre-mRNA (Eperon, 2012; Rigo et al., 2012; Singh and Cooper, 2012). These techniques were first designed to suppress the use of a particular pattern by blocking the binding of splicing factors to splice sites or exons (Dominski and Kole, 1993; Mayeda et al., 1990) and were subsequently developed as potential therapies for muscular dystrophy in cases where skipping of an exon carrying a nonsense mutation would be beneficial (Cirak et al., 2011; Dunckley et al., 1998; Goemans et al., 2011). The development of oligonucleotides that had the opposite effect, stimulating exon splicing, followed from the discovery of exonic splicing enhancers (ESEs). ESE sequences in the exons of normal pre-mRNA are bound by activator proteins, the best characterized of which are the SR proteins. These proteins contain one or two RNA-binding domains and a C-terminal domain rich in RS dipeptides. The C-terminal domain of an ESE-bound SR protein was proposed to interact directly with the 3′ splice site-recognition factor, U2AF, the recruitment of which is often a limiting step in splicing, thereby increasing the level of binding of U2AF (Graveley et al., 2001; Lavigueur et al., 1993; Staknis and Reed, 1994; Wang et al., 1995; Wu and Maniatis, 1993). This led to the development of two types of oligonucleotides to stimulate usage of an exon. In one version, a PNA sequence complementary to a target exon is attached to a short RS domain peptide (Cartegni and Krainer, 2003). In the other, an oligonucleotide complementary to a target exon is extended by an ESE sequence (Skordis et al., 2003). These bipartite oligonucleotides are referred to as targeted oligonucleotide enhancers of splicing (TOES) (Eperon and Muntoni, 2003). Other sequences in or around exons have been found to act as silencers, and in such cases activation can also be achieved by using oligonucleotides to block the binding of repressor proteins (Hua et al., 2007, 2008).

CrossMark

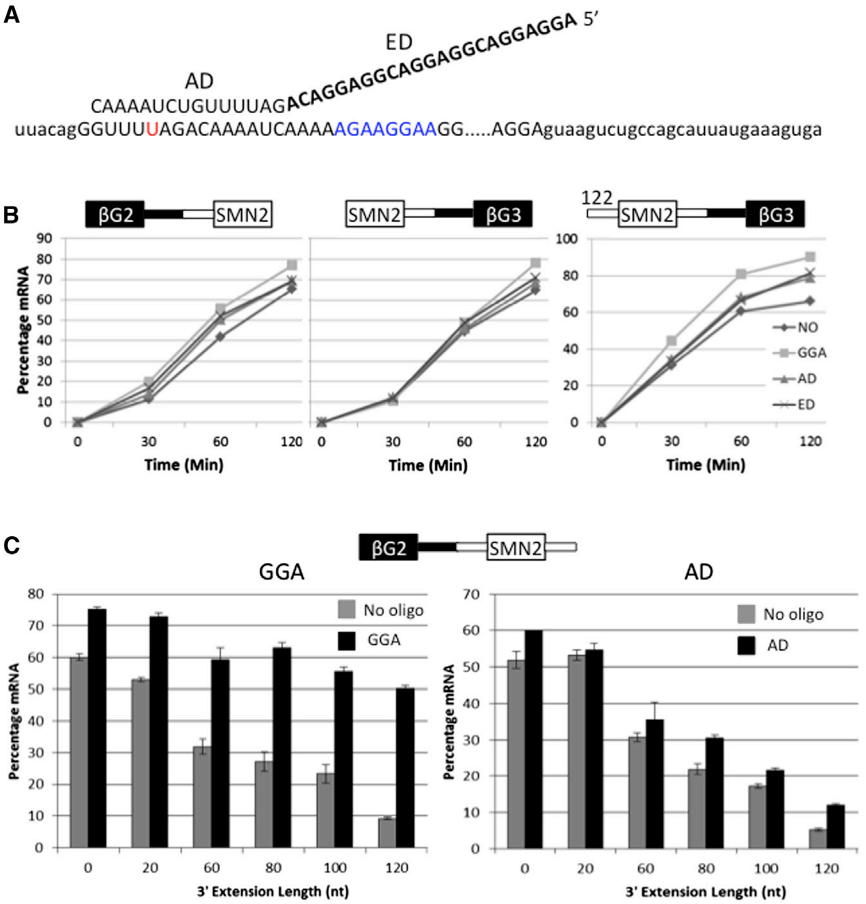

Figure 1. Inhibition of Splicing of SMN2 Exon 7 by Intron 7 Sequences and Activation by GGA

(A) Sequence of the TOES oligonucleotide GGA (AD, annealing domain; ED, ESE domain) annealed to SMN2 exon 7 (lowercase, intron; uppercase, exon [with the portion omitted shown by the dotted line]; red, site of the C/U difference in SMN1 versus SMN2; blue, exonic enhancer).

(B) Effects of oligonucleotides at 250 nM on the time courses of splicing in vitro of the introns 5′ and 3′ of exon 7 (SMN2) in SMN2/β-globin chimeric substrates (Owen et al., 2011; Skordis et al., 2003). The substrate in the right-hand panel included 122 nt of intron 6 preceding exon 7. NO, no oligonucleotide.

(C) Effects of increasing lengths of SMN2 intron 7 on the response of a β-globin/SMN2 exon 7 chimeric substrate to GGA and AD oligonucleotides. In vitro splicing assays were done in triplicate for 2 hr with oligonucleotides at 250 nM. Error bars show the SDs of the triplicates.

One of the difficulties in designing oligonucleotides that mimic the actions of ESEs and SR proteins is that the mechanisms of activation by the latter are still poorly understood (Eperon, 2012). In addition to the recruitment of U2AF to weak 3′ splice sites, the RS domains of ESE-bound SR proteins have also been shown to stabilize RNA duplexes formed between the 5′ splice site and branchpoint sequences with U6 and U2 snRNA, respectively (Shen and Green, 2006). One SR protein, SRSF1, enhances U1 snRNP binding to the 5′ splice site via protein interactions of its RRM domains (Cho et al., 2011). It is possible that some of these interactions may not be direct, since the introduction of a non-RNA linker between an ESE and a target 5′ splice site was shown to prevent ESE activity in vitro (Lewis et al., 2012), and not all of the interactions may be involved at every ESE or made by every SR protein. Therefore, it is difficult to identify the deficiencies in an exon's splicing signals and the best ways to compensate for them.

TOES oligonucleotides have been used to activate exons in SMN2 (Skordis et al., 2003; Marquis et al., 2007; Baughan et al., 2009), Ron (Ghigna et al., 2010), and IKBKAP (Ibrahim et al., 2007). Important determinants of the prototypical TOES oligonucleotide-activating SMN2 exon 7 include its site of annealing in the exon, the number of ESE-type motifs, and the inclusion of a non-RNA linker between the domains (Owen et al., 2011; Perrett et al., 2013). Surprisingly, previous we found that it augmented splicing equally effectively even in the absence of an ESE in exon 7 (Owen et al., 2011) that is otherwise essential (Hofmann et al., 2000; Martins de Araújo et al., 2009), the sites of annealing that enabled efficient activation of SMN2 exon 7 were very restricted, and stabilizing the annealing of the oligonucleotide with modified nucleotides reduced its activity (Owen et al., 2011). These findings suggest that the mechanistic models used as a basis for designing the oligonucleotides were inadequate, which compromises our ability to apply TOES oligonucleotides to rescue other exons. Consequently, we investigated the mechanisms by which the two domains of the oligonucleotide contribute to splicing activation.

RESULTS

Identification of Sequences that Suppress Exon 7 Splicing and Are Counteracted by GGA

The most effective TOES oligonucleotides tested on SMN2 anneal to exon 6 or 7 and contain three repeats of the sequence GGAGGAC in the ESE portion (Owen et al., 2011). Here, we focus on an oligonucleotide, termed GGA, that anneals to exon 7 (Figure 1A). This stimulates inclusion of exon 7 both in vitro and in fibroblasts derived from patients (Skordis et al., 2003); in the latter case, SMN protein expression is elevated for more than 28 days after a second transfection of cells with the oligonucleotide (Owen et al., 2011).

The standard pre-mRNA used to test the activity of TOES oligonucleotides contains SMN2 exon 7 and adjacent intron sequences flanked by portions of introns and exons of β-globin. The effects of TOES oligonucleotides on this pre-mRNA were previously shown to correlate well with those on the endogenous SMN2 gene in patient fibroblasts (Owen et al., 2011). To identify

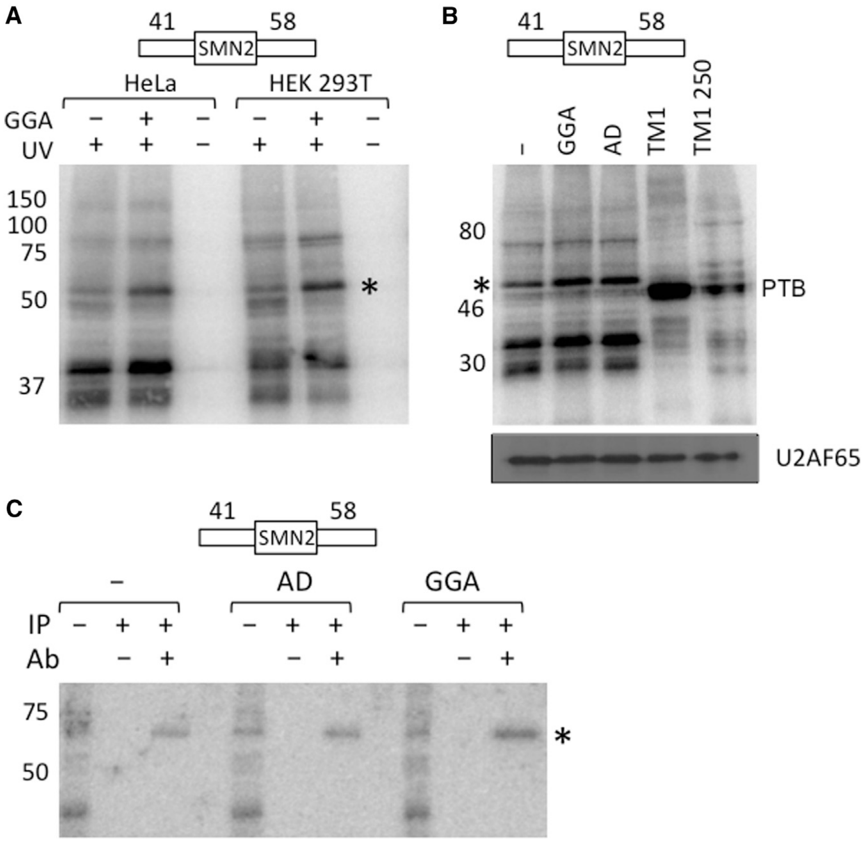

Figure 2. Effects of GGA and AD on U2AF65 Recruitment

(A) Effect of the GGA oligonucleotide on proteins crosslinking to SMN2 exon 7 in nuclear extracts. ^{32}P-uridine-labeled RNA comprising SMN2 exon 7 flanked by the 3′ 41 nt of intron 6 and the 5′ 58 nt of intron 7 was incubated in nuclear extracts from HeLa or 293T cells with 250 nM GGA. UV irradiation and RNase treatment were followed by SDS-PAGE and transfer to nitrocellulose. The positions of molecular weight markers are shown. The asterisk marks a band that increased in intensity when GGA was present.

(B) Effects of GGA or AD oligonucleotides on crosslinking of the SMN2 substrate to a protein that migrates with U2AF65. TM1 is a tropomyosin exon-based RNA that crosslinks very efficiently to PTB (Cherny et al., 2010), but at 250 mM KCl the PTB signal is reduced and a U2AF65 band just above it is detectable. After exposure, the membrane was incubated with antibodies to detect U2AF65. The U2AF bands coincide with the radioactive band marked with an asterisk.

(C) Immunoprecipitation after crosslinking with anti-U2AF65. IP−, total crosslinked mixture as in (A) and (B); Ab−, procedures done in the absence of antibody.

the splicing reactions stimulated by GGA, we tested transcripts containing single introns. The results (Figure 1B) showed that there was little stimulation of splicing. One possible explanation for this is that GGA is active when exon 7 is flanked by both introns 6 and 7. To test this, various lengths of a second intron were incorporated into the transcripts. GGA had little effect on splicing when exon 7 was preceded by the proximal 122 nt of intron 6 (Figure 1B). However, extending the length of intron 7 incorporated to the 3′ side of exon 7 progressively reduced the efficiency of splicing and GGA substantially counteracted this effect (Figure 1C): an oligonucleotide containing only the annealing domain (AD) had less effect than GGA, but still enhanced splicing slightly. We conclude that GGA counteracts the inhibitory effects of intron 7 on the splicing of intron 6.

Effects of the AD of GGA on ATP-Independent Binding of U2AF65

These results suggest that the oligonucleotide overcomes a limiting step in spliceosome assembly imposed in part by sequences in intron 7. Early steps of assembly involve the binding of factors at the 5′ and 3′ splice sites, which can be studied independently of subsequent events if ATP is omitted. The first factor known to bind specifically to 5′ splice sites is the U1 snRNP (Roca et al., 2013). Neither psoralen crosslinking nor immunoprecipitation revealed any effects of the oligonucleotide on the binding of U1 snRNP to the 5′ splice site of intron 7 (data not shown). The binding of protein factors was analyzed by UV crosslinking to a radiolabeled transcript comprising exon 7 flanked by 41 nt of intron 6 and 58 nt of intron 7. GGA and AD increased the levels of crosslinking to a band of 50–75 kDa, detected after transfer of the proteins onto a nitrocellulose filter (Figures 2A and 2B). Comparison with PTB crosslinked to a TPM1 substrate (Cherny et al., 2010) showed that the band comigrated with U2AF65, a protein required for the assembly of early splicing complexes that binds to 3′ss polypyrimidine tracts. Moreover, the radioactivity from crosslinking coincided perfectly with the chemiluminescent signal that arose from immunodetection of U2AF65 on the same filter (Figure 2B). Finally, immunoprecipitation of U2AF65 confirmed in both of two trials that GGA and, to a lesser extent, AD increased U2AF65 crosslinking to the transcript (Figure 2C). We conclude that GGA and AD facilitate the binding of U2AF65 to the 3′ss of intron 6.

Effects of the ESE Domain of GGA on the Formation of ATP-Dependent Complexes

We analyzed later stages of assembly by detecting heparin-resistant complexes that formed on the same exon 7-based transcript in the presence of ATP. GGA strongly promoted the assembly of a U2-dependent complex (ED-A; Figures 3A and 3B) and base-pairing of U2 snRNA to the transcript (Figure 3C). Oligonucleotide AD had a much smaller effect. A 2′-O-methyl oligonucleotide complementary to the 5′ end of U1 snRNA reduced ED-A formation, showing that U1 snRNP also contributed to assembly (data not shown). When we analyzed complex assembly on the intron 6-based transcript with 80 nt of intron 7 to the 3′ side (Figure 1C), we found that GGA stimulated assembly

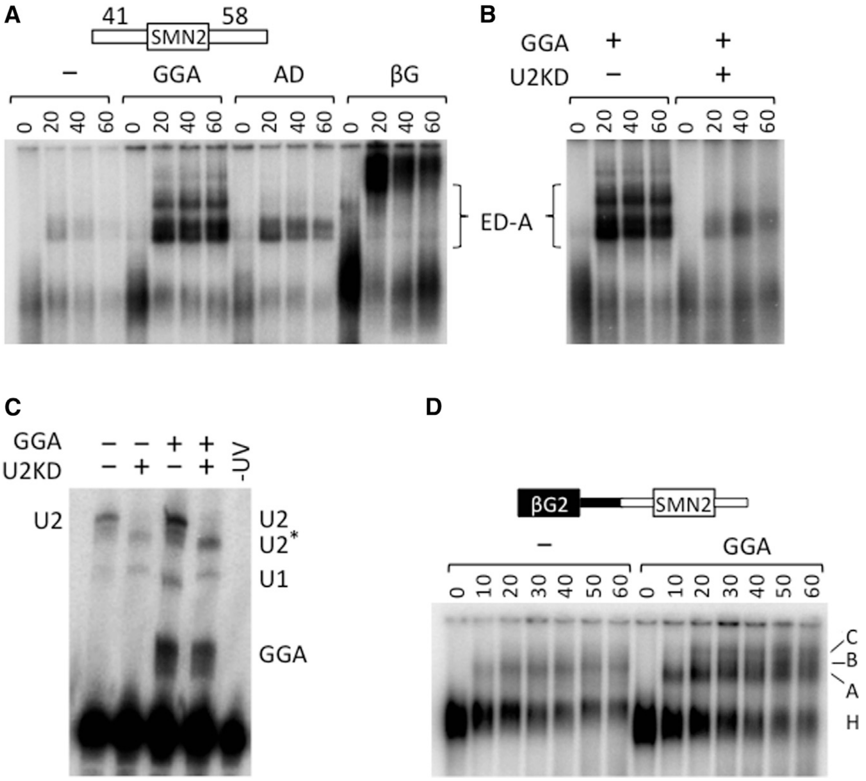

Figure 3. Effects of GGA and AD on Assembly of U2 snRNP-Containing Complexes

(A) Time courses of complex assembly in nuclear extract containing ATP on SMN2 exon 7 flanked by intron sequences as shown. As in Figure 2, GGA or AD oligonucleotides were included at 250 nM as shown; βG is a pre-mRNA derived from β-globin. The levels of radiolabeled RNA in the absence of oligonucleotide are usually much reduced, possibly due to degradation of RNA that does not enter spliceosome-related complexes. ED-A marks the exon-defined complex A.

(B) Effects on complex assembly of preincubation of extract with DNA oligonucleotides directing RNase H degradation at two sites of U2 snRNA.

(C) Psoralen-mediated crosslinking of radiolabeled SMN2 exon 7 substrate incubated in nuclear extract with GGA and/or oligonucleotides directing degradation of U2 snRNA. Adducts crosslinked to the substrate are indicated. The band labeled as U2* is presumed to be U2 snRNA that was cut only at the 5′ end and was still able to associate with the substrate.

(D) Stimulation by GGA of spliceosome assembly on pre-mRNA comprising β-globin exon 2/SMN2 exon 7 with 80 nt of intron 7 to the 3′ side.

of the prespliceosome (complex A) and spliceosomal complexes (Figure 3D). We conclude that whereas the AD is sufficient to stimulate binding of U2AF65 to the 3′ splice site of intron 6, the ESE domain of GGA stimulates the recruitment of U2 snRNPs and the assembly of complex A and spliceosomes.

Affinity Purification of GGA-Protein Complexes by Photoelution and Mass Spectrometry

To identify the proteins bound to GGA, we performed conventional affinity purification using a biotinylated transcript with the same sequence as GGA that had been incubated in nuclear extract. Proteins were separated by gel electrophoresis and bands were analyzed by mass spectrometry (MS). This revealed about eight major proteins and a number of proteins that were less abundant (data not shown). The most abundant proteins included hnRNP U, DHX36, nucleolin, hnRNP F/H, hnRNP A1, and CNBP, but not SRSF1. The high number of proteins recovered suggested that the transcript was forming heterogeneous complexes. One likely reason for this was that the concentration of the oligonucleotide was too high relative to the availability and affinity of specific proteins. Another possibility was that specific proteins, such as SRSF1, dissociated during purification and were replaced by other proteins. Native gel electrophoresis was used to test whether heterogeneous complexes formed (Figures S1A and S1B). A discrete complex formed in low concentrations of nuclear extract or in the presence of heparin, and several larger complexes formed in higher concentrations of nuclear extract and in the absence of heparin. We conclude that there is a core complex of tightly bound proteins formed on the ESE domain that is augmented or replaced by additional proteins in functional splicing conditions.

To establish that the isolated proteins came from the core complex, we sought a method that would enable intact complexes to be purified, eluted in native conditions, and analyzed by native gel electrophoresis. Moreover, it was important to identify the proteins that were associated with the full functional oligonucleotide but bound to the ESE domain. For this purpose, we performed affinity purification and photocleavage (Figure 4A). Complexes assembled on GGA were captured on avidin beads via a 3′ biotin adduct, and the ED complex was selectively eluted by cleavage of a photosensitive linkage between the AD and ED (Figures 4A and 4B). The biotinylated and photocleavable oligonucleotide assembled complexes similar to those formed on GGA, and cleavage after retention on avidin-agarose released a complex that comigrated with the complex formed on the ED (Figure S1C). Moreover, the photocleavable oligonucleotide stimulated exon 7 inclusion in splicing assays, and irradiation during the reaction reduced its efficacy (Figures S1D and S1E). MS on the proteins eluted after photocleavage produced a list of 41 proteins that were not in the control sample (Table S1). These were ranked based on the intensities of the top three peptides of each protein (Silva et al., 2006). The abundant proteins were clustered by relative molecular mass (Mr) into four groups: (1) a high-Mr group comprising helicase DHX36, exonuclease XRN2, nucleolin, and hnRNP U; (2) hnRNP F/H; (3) hnRNP A1 and SR proteins; and (4) CNBP. The proteins most likely to be responsible for the activity of the ESE domain in stimulating the recruitment of U2 snRNP and assembly of complex A are the SR proteins. Of these, SRSF1 was ranked most highly by MS (9th of 41 proteins), whereas the next most abundant, SRSF2,

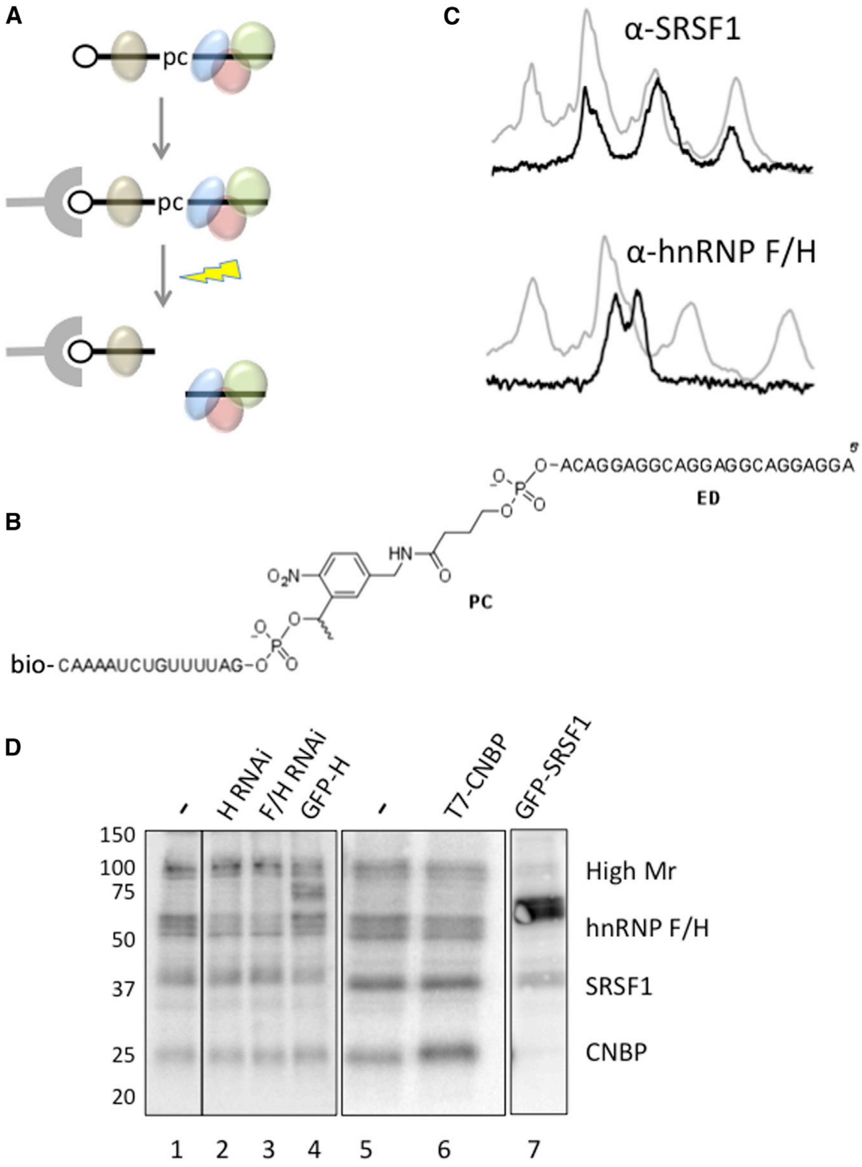

Figure 4. Binding of Proteins to the ESE Domain of GGA

(A) Strategy for selection by photoelution of proteins binding to the ED after assembly on the intact functional oligonucleotide GGA-PC-bio. Pc, photocleavable linkage; circle, biotin; gray, avidin beads; lightning bolt, irradiation with long-wave UV.

(B) Diagram of GGA-PC-bio.

(C) Crosslinking by short-wave UV to 5′ end-labeled ED in HEK293T extracts, followed by immunoprecipitation with anti-SRSF1 (left) or anti-hnRNP F/H (right). Phosphorimager profiles of the input (gray lines) and precipitated reactions (black lines) are shown from parallel gel lanes in each experiment. Ordinate, digital light units, arbitrary linear scale; abscissa, distance migrated, on an arbitrary linear scale with the origin on the left. Control lanes (minus antibody and minus irradiation) were blank in both experiments. The ordinate scale of the immunoprecipitations was expanded for visual comparison; for example, the signal from the immunoprecipitated SRSF1 was approximately 3% of the signal predicted from the input intensity.

(D) Crosslinking as in (C), without immunoprecipitation. Lanes 1–4 show results from one gel of crosslinking in functional nuclear extracts from cells transfected with siRNAs complementary to hnRNP H or F and H, as shown, or expressing GFP-hnRNP H; an intervening lane is not shown. Lanes 5–7 show results of crosslinking in extracts prepared from cells expressing T7-CNBP and GFP-SRSF1. The labels to the right indicate the identities of major components of each band. The bands in between the high-Mr proteins and hnRNP F/H correspond to GFP fusion proteins, as indicated.

See also Figure S1 and Tables S1 and S2.

ranked only 25th. The ESE domain was designed to recruit SRSF1, which is known to bind GGA motifs with high affinity (Tacke and Manley, 1995; Sanford et al., 2009; Cho et al., 2011; Cléry et al., 2013; Pandit et al., 2013; Ray et al., 2013), and we previously showed that recombinant SRSF1 is able to bind it specifically (Owen et al., 2011). The hnRNP proteins F and H bind G-triplets, and especially GGGA (Caputi and Zahler, 2001; Dominguez et al., 2010). G-triplets are generally found in introns, where they are associated with the stimulation of splicing by hnRNP H (Chou et al., 1999; Han et al., 2005; McCullough and Berget, 1997; Xiao et al., 2009). They are generally considered to be uncommon and associated with inhibition when found in exons (Chen et al., 1999; Lim et al., 2011; Mauger et al., 2008), although other findings suggest that sites within exons can also activate inclusion by recruiting hnRNP H (Caputi and Zahler, 2002; Huelga et al., 2012). The other groups of proteins are not generally considered to be splicing factors. Nucleolin, CNBP, and DHX36 are all associated with G-quadruplexes: CNBP binds single-stranded purine-rich sequences, such as GGA (Armas et al., 2008; Liu et al., 1998b; Michelotti et al., 1995), and promotes the formation of parallel G-quadruplexes (Borgognone et al., 2010); nucleolin binds and stabilizes parallel G-quadruplexes (Bates et al., 1999; González et al., 2009); and DHX36 binds tightly to G-quadruplexes and promotes unwinding (Creacy et al., 2008; Giri et al., 2011; Vaughn et al., 2005).

Different Sets of Proteins Contact the ESE Domain

To test for the presence of a common core complex, we incubated end-labeled ED at low nanomolar concentrations in nuclear extract and proteins crosslinked to it by short-wave UV. Potential components of the complex were immunoprecipitated. Since there was no treatment with ribonuclease, we expected that all of the crosslinked proteins would be detected in constant ratios regardless of the antigen selected if there were a single discrete complex. Without immunoprecipitation,

crosslinking showed, as expected, that the ED was in contact with the proteins of ~100, 50, 35, and 20 kDa, allowing for the mass of the attached oligonucleotide (Figures 4C and 4D). Similar results were found with 5′ end-labeled GGA attached to a photoreactive aryl azide and irradiated with long-wave UV (data not shown). However, immunoprecipitation with antibodies to SRSF1 and hnRNP F/H produced different patterns, demonstrating that there are different discrete complexes (Figure 4C). With anti-SRSF1, the high-Mr proteins were absent and the levels of hnRNP F/H and CNBP crosslinking were reduced 4-fold relative to SRSF1 (Figure 4C); with anti-hnRNP F/H, none of the 100 kDa, 30 kDa, or 20 kDa crosslinked proteins were recovered. This shows that in the majority of oligonucleotides where it was bound, hnRNP F/H excluded other proteins from making crosslinks to the RNA. In contrast, binding by SRSF1 was incompatible with high-Mr proteins, but not with hnRNP F/H or CNBP. The incompatibility of binding by SRSF1 with binding by DHX36 and nucleolin could be explained if the binding of the latter two proteins, unlike hnRNP F/H or CNBP, required the entire ESE domain. Alternatively, the oligonucleotide itself might exist in at least two different conformations: one bound by high-Mr proteins and one bound by hnRNP F/H, SRSF1, and CNBP in competition. The existence of competition among the high-Mr proteins, hnRNP F/H, and SRSF1 was tested by depletion of hnRNP F/ H by RNAi or overexpression of GFP-hnRNP H or GFP-SRSF1 (Figure 4D). The levels of crosslinking to hnRNP F/H were inversely correlated with those of crosslinking to the high-Mr proteins and SRSF1 (Table S2).

Formation of G-Quadruplexes by the ESE Domain

Since nucleolin and DHX36 bind quadruplexes, whereas the binding of hnRNP F/H is prevented by quadruplex formation (Samatanga et al., 2013), quadruplex formation could account for at least part of the heterogeneity. The ESE domain of GGA contains three GGAGGAC repeats (5′-AGGAGGACGGAGGAC GGAGGACA) (Owen et al., 2011). We synthesized the 23-mer as RNA and its 2′-OMe and 2′-OMe/PS derivatives, together with a truncated version, as a 15-mer RNA. Analysis by ^{1}H NMR at 800 MHz in 90% H_2O solution (Figure 5A) identified imino proton resonances at high ppm values (>9 ppm) that are characteristic of hydrogen-bonded NHs. All four natural and 2′-OMe RNA sequences also revealed a set of imino-NH resonances between 11 and 12 ppm. These are consistent with the existence of G-tetrads in a quadruplex. The poor resolution of these NH resonances in the 23-mers likely arises from the existence of multiple different quadruplex folds produced by the six GG motifs. Correspondingly, the spectrum of the 15-mer ESE with four GG motifs is better resolved.

The NMR data were confirmed by far-UV circular dichroism (CD) spectra (Figure 5B), which show the characteristic features of a stable, parallel folded, quadruplex structure, with a strong positive band at 260 nm and a weak negative band at 240 nm. However, the RNA versions show an additional atypical shoulder of positive ellipticity in the CD spectrum between 290 and 305 nm. In addition, thermal stability studies by CD (at ~260 nm) show significant hysteresis between the melting and refolding curves for the RNA, but not the modified oligonucleotides (Figure 5C; Table S3). The shoulder at 290–305 nm and the hysteresis are consistent with previous reports that GGAGG-based RNA sequences form end-to-end stacks of two quadruplex motifs via tetrad-hexad arrangements in which the adenosines are recruited to form the hexad and stabilize the dimer (Liu et al., 2002; Mashima et al., 2013; Uesugi et al., 2003). Electrospray ionization (ESI)-MS analysis of the 15-mer and 23-mer ESEs provides strong support for the formation of a dimeric structure (Figure 5D). To test whether the ESE domain formed a quadruplex structure in functional conditions, we performed primer extension assays in parallel on pure GGA and on GGA in a nuclear extract in splicing reaction conditions (Figure 5E). In both cases, pause sites were seen at the nucleotide prior to the first two 3′-GGA-5′ motifs in the template, but there was much reduced pausing prior to 3′-GGC-5′ motifs. This is consistent with a model in which AGG sequences participate in intermolecular G-quadruplexes even in nuclear extracts. The reduced intensity of the pause prior to the 5′-most AGG sequences could be accounted for by a propensity of the dimer to dissociate once the first of the four strands has been removed from the quadruplex by reverse transcription.

Contributions of Complexes Lacking SRSF1 to Exon 7 Inclusion

Our results showed that there are at least two major states of the oligonucleotide that are unlikely to contain SRSF1: a quadruplex dimer and the predominant hnRNP F/H complex. To test whether the quadruplex might contribute to activation of exon 7, we examined the effects of aromatic compounds that might stabilize quadruplexes. GSA-0902 and GSA-0802 are mono- and disubstituted quindolines, respectively, that bind DNA G-quadruplexes (Figure S2; Boddupally et al., 2012). CD was used to test whether these compounds stabilized the ESE RNA quadruplexes. The ligands induced changes in the quadruplex secondary structure of the 15-mer, but the spectra were consistent with the persistence of a predominantly parallel folded topology. The hysteresis in the melting and refolding curves persisted in the presence of the ligands, but the midpoints increased by 4°C−7°C, consistent with stabilization of the ligand-bound quadruplexes (data not shown). Splicing assays were done in triplicate with different concentrations of the quadruplex-binding reagents in the presence or absence of the GGA oligonucleotide (Figures 6A and 6B). In the presence of GGA, the majority of the spliced products included exon 7, but addition of the quadruplex stabilizers produced a dose-dependent decline in the level of inclusion. There was also a decline in the level of inclusion in the absence of GGA, but it was proportionately much smaller. We conclude that stabilizers, and therefore probably the quadruplexes themselves, do not contribute to and indeed compromise the activity of the bifunctional GGA oligonucleotide.

We tested the effect of hnRNP F/H on the activity of GGA after knockdown or overexpression of hnRNP F and H, using the extracts assayed for crosslinking to the ED (Figure 4D). The time courses of splicing showed very little effect on the proportion of SMN2 exon 7 inclusion, whether in the presence or absence of GGA (Figures 6C and S3), even though the reduction in levels of hnRNP F/H by RNAi was significant (Figure 6D).

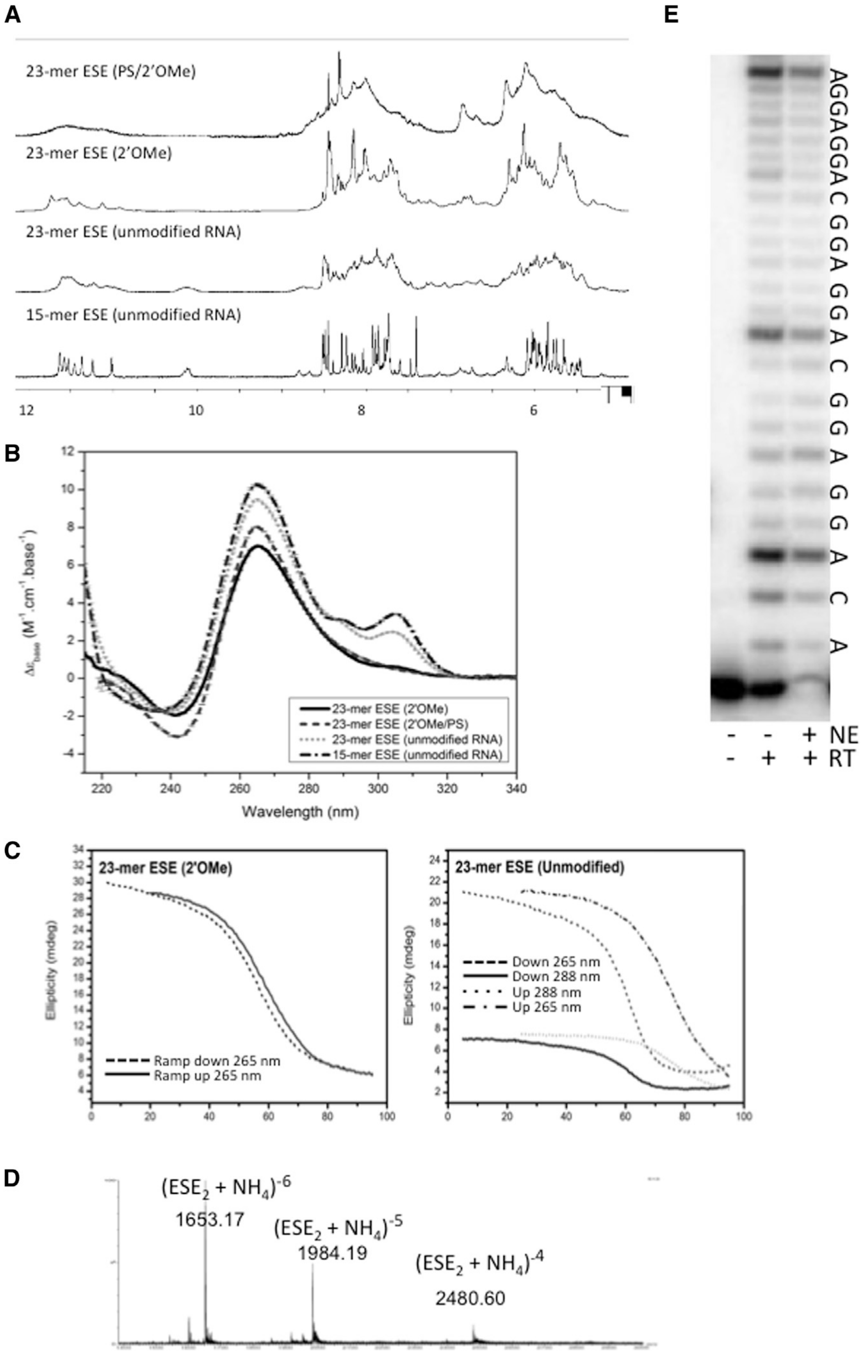

Figure 5. Formation of a Quadruplex by GGA

(A) Proton NMR spectra of the EDs synthesized from RNA (as in GGA) and 2′-OMe or phosphorothioate/2′-OMe nucleotides. The 15-mer is a truncated version of ED (ACGGAGGACGGAGGA).

(B) Far-UV CD spectra of ESE domains.

(C) Hysteresis in CD curves for melting and refolding of natural and backbone-modified ESE sequences. Oligonucleotides were at 1–6 μM and the temperature gradient was 3 min/0.5°C with 0.2°C tolerance. Spectra were recorded in 100 mM KCl, 10 mM K_2HPO_4/KH_2PO_4 at pH 7 with 10% D_2O.

(D) ESI mass spectrum of the 15-mer ESE (4956.1 $g.mol^{-1}$), 150 mM NH_4OAc, 10% MeOH.

(E) Primer extension on GGA in the presence or absence of nuclear extract. Extension from a primer annealed to the AD is shown from right to left; the template (GGA) sequence is also shown (5′ to left).

See also Table S3.

DISCUSSION

Here, we have described experiments addressing the effects of oligonucleotides that stimulate exon inclusion on the processes of splicing. This is important both because our findings will facilitate future applications of bifunctional oligonucleotides and because these oligonucleotides provide tools for investigating the actions of enhancers. We have shown that GGA stimulates splicing of the upstream intron, counteracting the negative influences of intron 7. In the absence of exogenous ATP, it enhances the formation of exon-defined complexes and the recruitment of U2AF65, and in the presence of ATP, it stimulates the formation of spliceosomal complexes and the recruitment of U2 snRNP. Moreover, we have shown that the enhancer (ESE) domain forms several mutually exclusive complexes in nuclear extracts and that it forms a quadruplex.

The deficiency of splicing of SMN2 exon 7 compared with SMN1 exon 7 is known to be caused by two sequence differences. The major one, a C-to-T transition in nt +6 of exon 7 (Lorson et al., 1999; Monani et al., 1999), converts a binding site for SRSF1 (Cartegni et al., 2006; Cartegni and Krainer, 2002) into a site for hnRNP A1 and/or Sam68 (Kashima and Manley, 2003; Kashima et al., 2007a, 2007b; Pedrotti et al., 2010). This results in a 2-fold reduced recruitment of U2AF65 as well as U2 snRNP, which depends on both U2AF65 and the central Tra2β-binding enhancer in the exon (Martins de Araújo et al., 2009). The contribution of an ESE to U2 snRNP binding is still generally thought to be mediated by U2AF, which is necessary and, at least in some circumstances, sufficient for U2 snRNP recruitment via interactions with SF3B1 (Mackereth et al., 2011; Ruskin et al., 1988; Valcárcel et al., 1996; Zamore and Green, 1989; Cass and Berglund, 2006; Gozani et al., 1998). However, there is also a U2AF-independent pathway for U2 recruitment (MacMillan et al., 1997) and ESEs can function without increasing U2AF binding (Li and Blencowe, 1999; Mühlemann et al., 2000), possibly to counteract silencers (Zhu and Krainer, 2000). The effects of AD on U2AF65 binding may explain the strong dependence of TOES activity on

OPEN ACCESS
CellPress

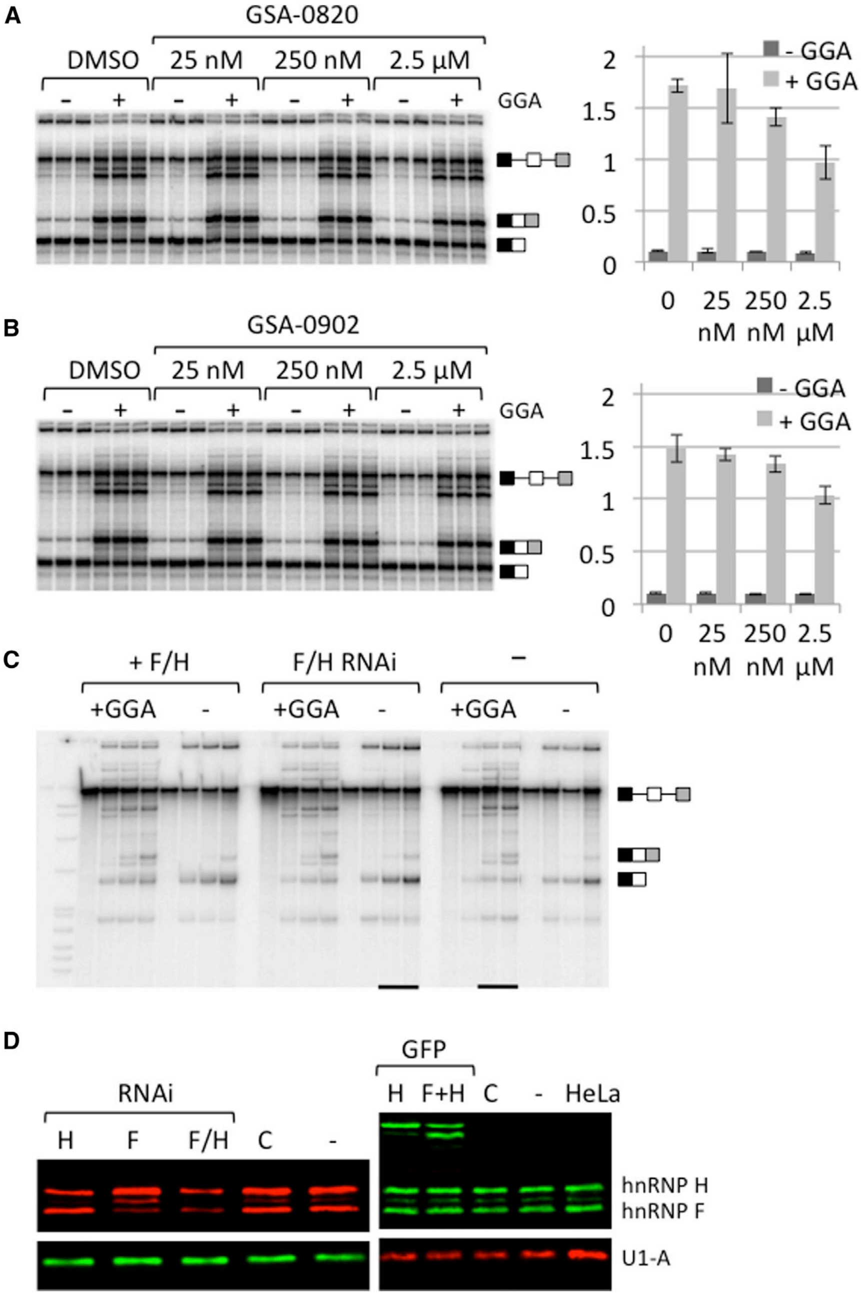

Figure 6. Contributions of the Quadruplex and hnRNP F/H to the Activity of GGA

(A and B) Triplicate assays of splicing in vitro of SMN2 exon 7-containing RNA were done in the presence or absence of 250 nM GGA. The quadruplex-stabilizing molecules GSA-0820 and GSA-0902 were dissolved in DMSO and added to the reactions to produce the final concentrations shown. The pre-mRNA and inclusion and exclusion mRNA products are indicated. Charts to the right of each phosphorimage show the ratio of inclusion/exclusion of exon 7 at each of the concentrations tested. Error bars show the SDs of the ratios for the triplicates.

(C) Time courses of splicing in vitro in the nuclear extracts used in Figure 4E. The extracts were prepared from HEK293T cells transfected with plasmids expressing mEGFP-hnRNP F and mEGFP-hnRNP H (+F/H) or siRNA targeting hnRNP F and H (F/H RNAi). −, mock transfection. Reactions were done in the presence or absence of GGA and samples taken at 0, 0, 60, and 120 min. The underlined lanes were transposed in the image.

(D) Analysis by western blotting of the nuclear extracts used for the experiments in Figures 4E and 6C. Antibodies detecting hnRNP F and H and U1A were detected by fluorescent secondary antibodies. C, mock transfection; −, untransfected. See also Figures S2 and S3.

the site of annealing within the exon (Owen et al., 2011), even though on its own it has only a very small effect on splicing even in the presence of the central ESE. GGA compensates for both of the deficiencies caused by the C-to-T mutation and also eliminates the dependence on the central ESE. In SMN1, the binding of SRSF1 to the 5′ end of the exon may contribute to U2 recruitment, whereas in SMN2 the ESE domain of GGA may provide sufficient contacts (Figure 7).

The inhibitory effect of intron 7 sequences on splicing of intron 6 and the lack of effect of oligonucleotides on the splicing of intron 6 in the absence of such sequences (Figure 1) suggest that the inhibitory effect of the C-to-T mutation is established in cooperation with intron 7 sequences. This might reflect a failure of an exon definition process, although exon definition currently lacks a mechanistic description and does not necessarily require more of the downstream intron than a 5′ splice site (Robberson et al., 1990; Kreivi et al., 1991). A possible explanation is that the known hnRNP A1 binding sites in intron 7 at nt +10 to +24 (Hua et al., 2008; Singh et al., 2006) and the SMN2-specific site at nt 100 (Kashima et al., 2007b), together with other likely sites in the exon (Vezain et al., 2010) and intron 7, may act in a concerted way to repress U2 snRNP recruitment. This may explain the need for additional contacts even when U2AF binding is observed.

The existence of quadruplex structures in functional conditions is generally difficult to establish, since they can be detected in short, pure RNA fragments but function within a longer RNA sequence in the presence of potential secondary structures and RNA-binding proteins. In contrast, GGA is a discrete functional unit that forms a quadruplex in isolation and also in nuclear extracts (Figure 5), where the structure antagonizes the activity of GGA (Figures 6A and 6B). The propensity to form quadruplexes may not be wholly disadvantageous. The head-to-head

OPEN
ACCESS
CellPress

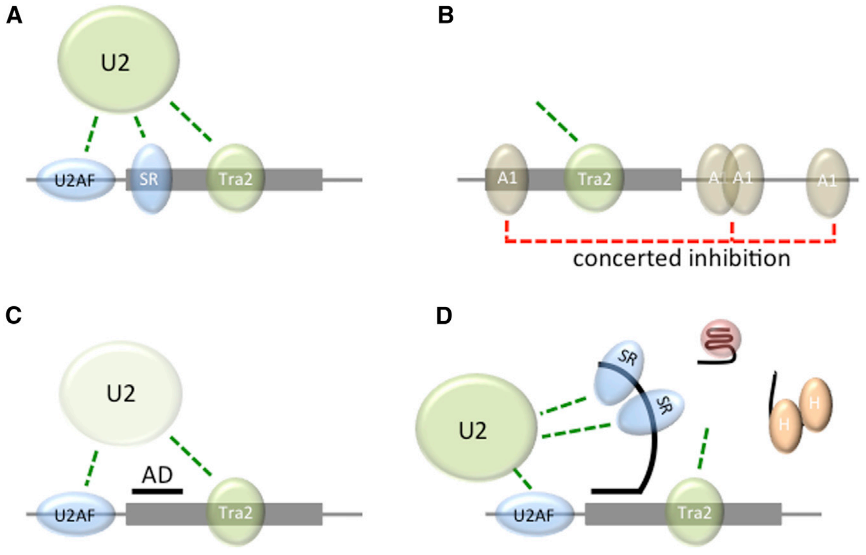

Figure 7. Models for the Effects of Oligonucleotides on Splicing of SMN2 Exon 7

(A) SMN1: SRSF1 bound to the 5′ end of the exon (Cartegni et al., 2006; Cartegni and Krainer, 2002), U2AF65 (Martins de Araújo et al., 2009), and aTra2β complex (Cléry et al., 2011; Hofmann et al., 2000) contribute to recruitment of U2 snRNP. Green dotted lines show putative functional interactions and may not correspond to direct molecular contacts.

(B) SMN2: binding of hnRNP A1 and other proteins (Kashima and Manley, 2003; Kashima et al., 2007a, 2007b; Pedrotti et al., 2010) to various sites are associated with loss of binding of SRSF1 and U2AF (Martins de Araújo et al., 2009) and U2 recruitment.

(C) The AD of GGA permits some U2AF binding, but U2 snRNP recruitment is weak.

(D) The GGA tail binds SRSF1 when the quadruplex is unwound, facilitating U2 recruitment independently of Tra2β. Nonproductive complexes of GGA with hnRNP F/H and in quadruplexes are also shown. Weak base-pairing or protein binding may enable rapid exchange of the various complexes on SMN2 exon 7 until an SRSF1 complex is stabilized by interactions with other proteins or the pre-mRNA.

dimers, in which tetrad-hexad quadruplexes are stacked together, may reduce the sensitivity of the oligonucleotide to 5′ nucleases. In addition, quadruplex formation is inimical to the binding of proteins that bind single-stranded RNA (Samatanga et al., 2013) and may reduce the sequestration of splicing activators by free GGA in the cell. This may be a generally useful property that could be exploited in the design of other functional oligonucleotides.

The other predominant form of GGA appeared to be a complex formed only with hnRNP F/H proteins, the binding of which is incompatible with quadruplex formation (Samatanga et al., 2013). SRSF1 complexes excluded nucleolin and DHX36 (Figure 4C) and thus are likely to also be in competition with quadruplexes. Overexpression of SRSF1 appeared to displace other proteins (Figure 4D), suggesting that there are multiple binding sites for SRSF1 on the ED, but that binding is not highly cooperative (Eperon et al., 2000), allowing partial occupancy and coexistence with other proteins. The coexistence of distinct complexes is likely to be a general feature of ESEs and may explain the common observation that SR proteins appear to be at best minor components of complexes when proteins bound to enhancers are analyzed after affinity purification (Staknis and Reed, 1994; Dreumont et al., 2010).

Competition between hnRNP F/H and SRSF1 may not, within limits, affect the efficacy of the oligonucleotide (Figures 4D and 6D; Table S2). Moreover, TOES activity was reduced by the use of SRSF1-binding sequences that would not bind hnRNP H (Owen et al., 2011). It is possible that the various forms of the oligonucleotide are in rapid equilibrium between pre-mRNA-bound and free states, enabling the rapid exchange of oligonucleotides in the quadruplex, hnRNP F/H, or SRSF1-containing complexes. A requirement for rapid oligonucleotide exchange would explain why increasing the strength of oligonucleotide base-pairing to exon 7 reduced TOES activity (Owen et al., 2011). Alternatively, there might be a rapid exchange of protein components bound to the oligonucleotide. Consistent with the idea that SR proteins have only a transient association with some enhancers are (1) the difficulties of demonstrating that SR proteins are the predominant proteins bound to enhancers (Staknis and Reed, 1994; Dreumont et al., 2010), (2) the inverse correlation found between SRSF2's affinity for an enhancer sequence and the activity of the enhancer (Dreumont et al., 2010), (3) the poor correlation between the more highly occupied SRSF1 and SRSF2 binding sites transcriptome-wide and their effects on splicing (Pandit et al., 2013), and (4) the increase in TOES activity with increased numbers of binding motifs (Owen et al., 2011). Moreover, functional targets for SRSF1 are more diverse than optimal binding sites, since half of them have neither a GGA nor a GAA motif (Liu et al., 1998a; Smith et al., 2006). The strength of binding of SRSF1 is likely to be crucial in those cases where its activity requires only the second RNA-binding domain, possibly because it displaces other RNA-binding proteins (Cléry et al., 2013). For the majority of enhancers, though, it may be that their activity does not depend on the strength of their binding to SR proteins and instead is based on fleeting "kiss and tell" encounters with SR proteins.

EXPERIMENTAL PROCEDURES

In Vitro Splicing and Related Assays

Splicing assays were done in nuclear extract (Cilbiotech) as previously described (Eperon and Krainer, 1994; Skordis et al., 2003). Complex formation on exon-defined transcripts or pre-mRNA in the presence or absence of exogenous ATP was analyzed as previously described (Das and Reed, 1999). Direct crosslinking in nuclear extracts with short-wave UV light was done as previously described (Eperon et al., 2000) using RNA transcribed with [α-^{32}P] UTP. Immunoprecipitation after crosslinking and digestion with ribonucleases A and T1 was done with an antibody to U2AF65 (Gama-Carvalho et al., 2006). Psoralen-mediated crosslinking of RNA to snRNA and ribonuclease digestion

were performed as previously described (O'Mullane and Eperon, 1998). TOES oligonucleotides were annealed to the RNA by incubation for 5 min at 80°C in 50 mM potassium glutamate, 20 mM HEPES (pH 7.5), followed by gradual cooling to 4°C. GGA, ED, and AD oligonucleotides have been described elsewhere (Owen et al., 2011). GSA-0820 and GSA-0902, dissolved in 10% DMSO, were included at 1/10th volume in annealing reactions consisting of GGA (500 nM), pre-mRNA, 100 mM potassium glutamate, and 40 mM HEPES (pH 7.5) in silanized plasticware. Incubation was done for 10 min at 30°C. Splicing reactions were initiated by addition of an equal volume of the remaining components and incubated at 30°C for 2 hr. Knockdown of hnRNPs F and H1 was done in HEK293T cells in 15 cm dishes. Knockdown of each hnRNP was done using two siRNAs (s230280 and s6725 for hnRNP F, and s6729 and s6730 for hnRNP H1; Ambion Life Technologies). Each knockdown was done using three successive transfections at 10 μM with Lipofectamine at intervals of 24 hr. Extracts were prepared as previously described (Lee et al., 1988).

Affinity Purification and Analysis of Complexes on Oligonucleotides

For conventional biotin affinity purification (Figure 4A), RNA corresponding to GGA was transcribed in the presence of biotin-11-UTP. Then 100 pmole of RNA was selected on avidin-agarose beads and incubated for 10 min in 200 μl nuclear extract under splicing reaction conditions. The beads were washed four times with 600 μl of ice-cold FSP buffer (20 mM Tris-HCl [pH 7.5], 60 mM KCl, 2.5 mM EDTA, 0.1% NP40) and once with FSP buffer without KCl, and bound proteins were eluted with a solution containing 2% SDS and 20 mM dithiothreitol. The GGA-PC-Bio oligonucleotide was synthesized by DNA Technology. For the purification of complexes, GGA-PC-Bio was incubated in nuclear extract in darkness and then the mixture was captured by incubation for 2–12 hr at 4°C with NeutrAvidin agarose (Thermo Scientific). After washing with 100 mM $NaPO_4$ [pH 7.2], 150 mM NaCl, 0.05% NP-40, and then 100 mM $NaPO_4$ [pH 7.2], 0.05% NP-40, the oligonucleotide was cleaved between the ED and AD by irradiation of moist beads with broad-range UV (UVP Bio-Lite) for 2.5 min. The ED complex was recovered by elution with 100 mM $NaPO_4$ [pH 7.2], 0.05% NP-40. Irradiation and elution were repeated thrice. Analysis by MS is described in the Supplemental Experimental Procedures. Crosslinking of GGA to associated proteins was done in two ways. In one, a *p*-azidotetrafluorobenzoic moiety was conjugated to the 5′ phosphate group of the ^{32}P-labeled GGA RNA via an ethylenediamine spacer as previously described (Malygin et al., 1999). After incubation of the RNA derivative (20 pmol) in 40 μl of nuclear extract under splicing reaction conditions and irradiation with long-wave UV light (>300 nm), the reaction mixture was treated with RNase A and analyzed by SDS-PAGE. Alternatively, the GGA or ED was 5′ end-labeled with ^{32}P, incubated in nuclear extracts prepared as previously described (Hodson et al., 2012), irradiated with short-wave UV light, and subjected to SDS-PAGE. Immunoprecipitation prior to electrophoresis was done with an antibody to SRSF1 (Hanamura et al., 1998) or hnRNP F/H (Abcam ab10689). The structural analysis of the oligonucleotides is described in the Supplemental Experimental Procedures.

SUPPLEMENTAL INFORMATION

Supplemental Information includes Supplemental Experimental Procedures, three figures, and three tables and can be found with this article online at http://dx.doi.org/10.1016/j.celrep.2014.08.051.

AUTHOR CONTRIBUTIONS

L.D.S. did the experiments identifying sequences required for GGA responsiveness, assays of splicing complex assembly, and UV crosslinking. R.L.D. devised and did the affinity purification with a photocleavable linker, assays of complex formation on the oligonucleotides, and splicing assays with quadruplex binders. C.M.L. prepared depleted extracts and did the crosslinking-immunoprecipitation and splicing assays in them. A.C. did NMR, CD, and MS experiments. A.A.M. did conventional biotin affinity purifications. C.W. did the depletions by RNAi. A.J.P. synthesized the RNA and modified the oligonucleotides for physical measurements. A.R.B. did the MS and analyzed the results. M.S.S. suggested, designed, and supervised the physical measurements. G.A.B. and I.C.E. devised the project and designed and supervised the chemical and biochemical aspects, respectively. L.D.S., A.R.B., M.S.S., and I.C.E. wrote the manuscript.

ACKNOWLEDGMENTS

We thank Dr. L. Hurley (University of Arizona) for the gift of quadruplex-stabilizing reagents, Professor M. Carmo-Fonseca (IMM, Lisbon) for the anti-U2AF65 antibody, and Dr. A.R. Krainer (Cold Spring Harbor Laboratory) for the anti-SRSF1 antibody. We also thank Ms S. Ashra (University of Leicester) and Ms R. Jukes-Jones and Prof. K. Cain (MRC Toxicology Unit) for assistance with MS. This work was supported by the Leverhulme Trust (grant F/00 212Y to G.A.B. and I.C.E.), the MRC (postgraduate studentship to L.D.S.), and the Association for International Cancer Research (M.S.S. and A.C.).

Received: April 22, 2013
Revised: July 17, 2014
Accepted: August 21, 2014
Published: September 25, 2014

REFERENCES

Armas, P., Nasif, S., and Calcaterra, N.B. (2008). Cellular nucleic acid binding protein binds G-rich single-stranded nucleic acids and may function as a nucleic acid chaperone. J. Cell. Biochem. *103*, 1013–1036.

Bates, P.J., Kahlon, J.B., Thomas, S.D., Trent, J.O., and Miller, D.M. (1999). Antiproliferative activity of G-rich oligonucleotides correlates with protein binding. J. Biol. Chem. *274*, 26369–26377.

Baughan, T.D., Dickson, A., Osman, E.Y., and Lorson, C.L. (2009). Delivery of bifunctional RNAs that target an intronic repressor and increase SMN levels in an animal model of spinal muscular atrophy. Hum. Mol. Genet. *18*, 1600–1611.

Boddupally, P.V., Hahn, S., Beman, C., De, B., Brooks, T.A., Gokhale, V., and Hurley, L.H. (2012). Anticancer activity and cellular repression of c-MYC by the G-quadruplex-stabilizing 11-piperazinylquindoline is not dependent on direct targeting of the G-quadruplex in the c-MYC promoter. J. Med. Chem. *55*, 6076–6086.

Borgognone, M., Armas, P., and Calcaterra, N.B. (2010). Cellular nucleic-acid-binding protein, a transcriptional enhancer of c-Myc, promotes the formation of parallel G-quadruplexes. Biochem. J. *428*, 491–498.

Caputi, M., and Zahler, A.M. (2001). Determination of the RNA binding specificity of the heterogeneous nuclear ribonucleoprotein (hnRNP) H/H'/F/2H9 family. J. Biol. Chem. *276*, 43850–43859.

Caputi, M., and Zahler, A.M. (2002). SR proteins and hnRNP H regulate the splicing of the HIV-1 tev-specific exon 6D. EMBO J. *21*, 845–855.

Cartegni, L., and Krainer, A.R. (2002). Disruption of an SF2/ASF-dependent exonic splicing enhancer in SMN2 causes spinal muscular atrophy in the absence of SMN1. Nat. Genet. *30*, 377–384.

Cartegni, L., and Krainer, A.R. (2003). Correction of disease-associated exon skipping by synthetic exon-specific activators. Nat. Struct. Biol. *10*, 120–125.

Cartegni, L., Hastings, M.L., Calarco, J.A., de Stanchina, E., and Krainer, A.R. (2006). Determinants of exon 7 splicing in the spinal muscular atrophy genes, SMN1 and SMN2. Am. J. Hum. Genet. *78*, 63–77.

Cass, D.M., and Berglund, J.A. (2006). The SF3b155 N-terminal domain is a scaffold important for splicing. Biochemistry *45*, 10092–10101.

Chen, C.D., Kobayashi, R., and Helfman, D.M. (1999). Binding of hnRNP H to an exonic splicing silencer is involved in the regulation of alternative splicing of the rat beta-tropomyosin gene. Genes Dev. *13*, 593–606.

Cherny, D., Gooding, C., Eperon, G.E., Coelho, M.B., Bagshaw, C.R., Smith, C.W., and Eperon, I.C. (2010). Stoichiometry of a regulatory splicing complex revealed by single-molecule analyses. EMBO J. *29*, 2161–2172.

Cho, S., Hoang, A., Sinha, R., Zhong, X.Y., Fu, X.D., Krainer, A.R., and Ghosh, G. (2011). Interaction between the RNA binding domains of Ser-Arg splicing

factor 1 and U1-70K snRNP protein determines early spliceosome assembly. Proc. Natl. Acad. Sci. USA *108*, 8233–8238.

Chou, M.Y., Rooke, N., Turck, C.W., and Black, D.L. (1999). hnRNP H is a component of a splicing enhancer complex that activates a c-src alternative exon in neuronal cells. Mol. Cell. Biol. *19*, 69–77.

Cirak, S., Arechavala-Gomeza, V., Guglieri, M., Feng, L., Torelli, S., Anthony, K., Abbs, S., Garralda, M.E., Bourke, J., Wells, D.J., et al. (2011). Exon skipping and dystrophin restoration in patients with Duchenne muscular dystrophy after systemic phosphorodiamidate morpholino oligomer treatment: an open-label, phase 2, dose-escalation study. Lancet *378*, 595–605.

Cléry, A., Jayne, S., Benderska, N., Dominguez, C., Stamm, S., and Allain, F.H. (2011). Molecular basis of purine-rich RNA recognition by the human SR-like protein Tra2-β1. Nat. Struct. Mol. Biol. *18*, 443–450.

Cléry, A., Sinha, R., Anczuków, O., Corrionero, A., Moursy, A., Daubner, G.M., Valcárcel, J., Krainer, A.R., and Allain, F.H. (2013). Isolated pseudo-RNA-recognition motifs of SR proteins can regulate splicing using a noncanonical mode of RNA recognition. Proc. Natl. Acad. Sci. USA *110*, E2802–E2811.

Creacy, S.D., Routh, E.D., Iwamoto, F., Nagamine, Y., Akman, S.A., and Vaughn, J.P. (2008). G4 resolvase 1 binds both DNA and RNA tetramolecular quadruplex with high affinity and is the major source of tetramolecular quadruplex G4-DNA and G4-RNA resolving activity in HeLa cell lysates. J. Biol. Chem. *283*, 34626–34634.

Das, R., and Reed, R. (1999). Resolution of the mammalian E complex and the ATP-dependent spliceosomal complexes on native agarose mini-gels. RNA *5*, 1504–1508.

Djebali, S., Davis, C.A., Merkel, A., Dobin, A., Lassmann, T., Mortazavi, A., Tanzer, A., Lagarde, J., Lin, W., Schlesinger, F., et al. (2012). Landscape of transcription in human cells. Nature *489*, 101–108.

Dominguez, C., Fisette, J.F., Chabot, B., and Allain, F.H. (2010). Structural basis of G-tract recognition and encaging by hnRNP F quasi-RRMs. Nat. Struct. Mol. Biol. *17*, 853–861.

Dominski, Z., and Kole, R. (1993). Restoration of correct splicing in thalassemic pre-mRNA by antisense oligonucleotides. Proc. Natl. Acad. Sci. USA *90*, 8673–8677.

Dreumont, N., Hardy, S., Behm-Ansmant, I., Kister, L., Branlant, C., Stévenin, J., and Bourgeois, C.F. (2010). Antagonistic factors control the unproductive splicing of SC35 terminal intron. Nucleic Acids Res. *38*, 1353–1366.

Dunckley, M.G., Manoharan, M., Villiet, P., Eperon, I.C., and Dickson, G. (1998). Modification of splicing in the dystrophin gene in cultured Mdx muscle cells by antisense oligoribonucleotides. Hum. Mol. Genet. *7*, 1083–1090.

Eperon, I. (2012). Antisense therapeutics: New ways to nudge splicing. Nat. Chem. Biol. *8*, 507–508.

Eperon, I.C., and Krainer, A.R. (1994). Splicing of mRNA precursors in mammalian cells. In RNA Processing: A Practical Approach, S.J. Higgins and B.D. Hames, eds. (Oxford, UK: IRL Press), pp. 57–101.

Eperon, I.C., and Muntoni, F. (2003). Response to Buratti et al.: Can a 'patch' in a skipped exon make the pre-mRNA splicing machine run better? Trends Mol. Med. *9*, 233–234.

Eperon, I.C., Makarova, O.V., Mayeda, A., Munroe, S.H., Cáceres, J.F., Hayward, D.G., and Krainer, A.R. (2000). Selection of alternative 5′ splice sites: role of U1 snRNP and models for the antagonistic effects of SF2/ASF and hnRNP A1. Mol. Cell. Biol. *20*, 8303–8318.

Gama-Carvalho, M., Barbosa-Morais, N.L., Brodsky, A.S., Silver, P.A., and Carmo-Fonseca, M. (2006). Genome-wide identification of functionally distinct subsets of cellular mRNAs associated with two nucleocytoplasmic-shuttling mammalian splicing factors. Genome Biol. *7*, R113.

Ghigna, C., De Toledo, M., Bonomi, S., Valacca, C., Gallo, S., Apicella, M., Eperon, I., Tazi, J., and Biamonti, G. (2010). Pro-metastatic splicing of Ron proto-oncogene mRNA can be reversed: therapeutic potential of bifunctional oligonucleotides and indole derivatives. RNA Biol. *7*, 495–503.

Giri, B., Smaldino, P.J., Thys, R.G., Creacy, S.D., Routh, E.D., Hantgan, R.R., Lattmann, S., Nagamine, Y., Akman, S.A., and Vaughn, J.P. (2011). G4 resolvase 1 tightly binds and unwinds unimolecular G4-DNA. Nucleic Acids Res. *39*, 7161–7178.

Goemans, N.M., Tulinius, M., van den Akker, J.T., Burm, B.E., Ekhart, P.F., Heuvelmans, N., Holling, T., Janson, A.A., Platenburg, G.J., Sipkens, J.A., et al. (2011). Systemic administration of PRO051 in Duchenne's muscular dystrophy. N. Engl. J. Med. *364*, 1513–1522.

González, V., Guo, K., Hurley, L., and Sun, D. (2009). Identification and characterization of nucleolin as a c-myc G-quadruplex-binding protein. J. Biol. Chem. *284*, 23622–23635.

Gozani, O., Potashkin, J., and Reed, R. (1998). A potential role for U2AF-SAP 155 interactions in recruiting U2 snRNP to the branch site. Mol. Cell. Biol. *18*, 4752–4760.

Graveley, B.R., Hertel, K.J., and Maniatis, T. (2001). The role of U2AF35 and U2AF65 in enhancer-dependent splicing. RNA *7*, 806–818.

Han, K., Yeo, G., An, P., Burge, C.B., and Grabowski, P.J. (2005). A combinatorial code for splicing silencing: UAGG and GGGG motifs. PLoS Biol. *3*, e158.

Hanamura, A., Cáceres, J.F., Mayeda, A., Franza, B.R., Jr., and Krainer, A.R. (1998). Regulated tissue-specific expression of antagonistic pre-mRNA splicing factors. RNA *4*, 430–444.

Hodson, M.J., Hudson, A.J., Cherny, D., and Eperon, I.C. (2012). The transition in spliceosome assembly from complex E to complex A purges surplus U1 snRNPs from alternative splice sites. Nucleic Acids Res. *40*, 6850–6862.

Hofmann, Y., Lorson, C.L., Stamm, S., Androphy, E.J., and Wirth, B. (2000). Htra2-beta 1 stimulates an exonic splicing enhancer and can restore full-length SMN expression to survival motor neuron 2 (SMN2). Proc. Natl. Acad. Sci. USA *97*, 9618–9623.

Hua, Y., Vickers, T.A., Baker, B.F., Bennett, C.F., and Krainer, A.R. (2007). Enhancement of SMN2 exon 7 inclusion by antisense oligonucleotides targeting the exon. PLoS Biol. *5*, e73.

Hua, Y., Vickers, T.A., Okunola, H.L., Bennett, C.F., and Krainer, A.R. (2008). Antisense masking of an hnRNP A1/A2 intronic splicing silencer corrects SMN2 splicing in transgenic mice. Am. J. Hum. Genet. *82*, 834–848.

Huelga, S.C., Vu, A.Q., Arnold, J.D., Liang, T.Y., Liu, P.P., Yan, B.Y., Donohue, J.P., Shiue, L., Hoon, S., Brenner, S., et al. (2012). Integrative genome-wide analysis reveals cooperative regulation of alternative splicing by hnRNP proteins. Cell Rep *1*, 167–178.

Ibrahim, E.C., Hims, M.M., Shomron, N., Burge, C.B., Slaugenhaupt, S.A., and Reed, R. (2007). Weak definition of IKBKAP exon 20 leads to aberrant splicing in familial dysautonomia. Hum. Mutat. *28*, 41–53.

Kashima, T., and Manley, J.L. (2003). A negative element in SMN2 exon 7 inhibits splicing in spinal muscular atrophy. Nat. Genet. *34*, 460–463.

Kashima, T., Rao, N., David, C.J., and Manley, J.L. (2007a). hnRNP A1 functions with specificity in repression of SMN2 exon 7 splicing. Hum. Mol. Genet. *16*, 3149–3159.

Kashima, T., Rao, N., and Manley, J.L. (2007b). An intronic element contributes to splicing repression in spinal muscular atrophy. Proc. Natl. Acad. Sci. USA *104*, 3426–3431.

Kreivi, J.P., Zerivitz, K., and Akusjärvi, G. (1991). A U1 snRNA binding site improves the efficiency of in vitro pre-mRNA splicing. Nucleic Acids Res. *19*, 6956.

Lavigueur, A., La Branche, H., Kornblihtt, A.R., and Chabot, B. (1993). A splicing enhancer in the human fibronectin alternate ED1 exon interacts with SR proteins and stimulates U2 snRNP binding. Genes Dev. *7* (12A), 2405–2417.

Lee, K.A., Bindereif, A., and Green, M.R. (1988). A small-scale procedure for preparation of nuclear extracts that support efficient transcription and pre-mRNA splicing. Gene Anal. Tech. *5*, 22–31.

Lewis, H., Perrett, A.J., Burley, G.A., and Eperon, I.C. (2012). An RNA splicing enhancer that does not act by looping. Angew. Chem. Int. Ed. Engl. *51*, 9800–9803.

Li, Y., and Blencowe, B.J. (1999). Distinct factor requirements for exonic splicing enhancer function and binding of U2AF to the polypyrimidine tract. J. Biol. Chem. *274*, 35074–35079.

Lim, K.H., Ferraris, L., Filloux, M.E., Raphael, B.J., and Fairbrother, W.G. (2011). Using positional distribution to identify splicing elements and predict pre-mRNA processing defects in human genes. Proc. Natl. Acad. Sci. USA *108*, 11093–11098.

Liu, H.X., Zhang, M., and Krainer, A.R. (1998a). Identification of functional exonic splicing enhancer motifs recognized by individual SR proteins. Genes Dev. *12*, 1998–2012.

Liu, M., Kumar, K.U., Pater, M.M., and Pater, A. (1998b). Identification and characterization of a JC virus pentanucleotide repeat element binding protein: cellular nucleic acid binding protein. Virus Res. *58*, 73–82.

Liu, H., Matsugami, A., Katahira, M., and Uesugi, S. (2002). A dimeric RNA quadruplex architecture comprised of two G:G(:A):G:G(:A) hexads, G:G:G:G tetrads and UUUU loops. J. Mol. Biol. *322*, 955–970.

Lorson, C.L., Hahnen, E., Androphy, E.J., and Wirth, B. (1999). A single nucleotide in the SMN gene regulates splicing and is responsible for spinal muscular atrophy. Proc. Natl. Acad. Sci. USA *96*, 6307–6311.

Mackereth, C.D., Madl, T., Bonnal, S., Simon, B., Zanier, K., Gasch, A., Rybin, V., Valcárcel, J., and Sattler, M. (2011). Multi-domain conformational selection underlies pre-mRNA splicing regulation by U2AF. Nature *475*, 408–411.

MacMillan, A.M., McCaw, P.S., Crispino, J.D., and Sharp, P.A. (1997). SC35-mediated reconstitution of splicing in U2AF-depleted nuclear extract. Proc. Natl. Acad. Sci. USA *94*, 133–136.

Malygin, A.A., Dobrikov, M.I., Repkova, M.N., Shishkin, G.V., Ven'yaminova, A.G., and Karpova, G.G. (1999). Proteins neighboring 18S rRNA conserved sequences 609-618 and 1047-1061 within the 40S human ribosomal subunit. RNA *5*, 1656–1664.

Marquis, J., Meyer, K., Angehrn, L., Kämpfer, S.S., Rothen-Rutishauser, B., and Schümperli, D. (2007). Spinal muscular atrophy: SMN2 pre-mRNA splicing corrected by a U7 snRNA derivative carrying a splicing enhancer sequence. Mol. Ther. *15*, 1479–1486.

Martins de Araújo, M., Bonnal, S., Hastings, M.L., Krainer, A.R., and Valcárcel, J. (2009). Differential 3′ splice site recognition of SMN1 and SMN2 transcripts by U2AF and U2 snRNP. RNA *15*, 515–523.

Mashima, T., Nishikawa, F., Kamatari, Y.O., Fujiwara, H., Saimura, M., Nagata, T., Kodaki, T., Nishikawa, S., Kuwata, K., and Katahira, M. (2013). Anti-prion activity of an RNA aptamer and its structural basis. Nucleic Acids Res. *41*, 1355–1362.

Mauger, D.M., Lin, C., and Garcia-Blanco, M.A. (2008). hnRNP H and hnRNP F complex with Fox2 to silence fibroblast growth factor receptor 2 exon IIIc. Mol. Cell. Biol. *28*, 5403–5419.

Mayeda, A., Hayase, Y., Inoue, H., Ohtsuka, E., and Ohshima, Y. (1990). Surveyingcis-acting sequences of pre-mRNA by adding antisense 2′-O-methyl oligoribonucleotides to a splicing reaction. J. Biochem. *108*, 399–405.

McCullough, A.J., and Berget, S.M. (1997). G triplets located throughout a class of small vertebrate introns enforce intron borders and regulate splice site selection. Mol. Cell. Biol. *17*, 4562–4571.

Melamud, E., and Moult, J. (2009). Stochastic noise in splicing machinery. Nucleic Acids Res. *37*, 4873–4886.

Michelotti, E.F., Tomonaga, T., Krutzsch, H., and Levens, D. (1995). Cellular nucleic acid binding protein regulates the CT element of the human c-myc protooncogene. J. Biol. Chem. *270*, 9494–9499.

Monani, U.R., Lorson, C.L., Parsons, D.W., Prior, T.W., Androphy, E.J., Burghes, A.H., and McPherson, J.D. (1999). A single nucleotide difference that alters splicing patterns distinguishes the SMA gene SMN1 from the copy gene SMN2. Hum. Mol. Genet. *8*, 1177–1183.

Mühlemann, O., Yue, B.G., Petersen-Mahrt, S., and Akusjärvi, G. (2000). A novel type of splicing enhancer regulating adenovirus pre-mRNA splicing. Mol. Cell. Biol. *20*, 2317–2325.

O'Mullane, L., and Eperon, I.C. (1998). The pre-mRNA 5′ cap determines whether U6 small nuclear RNA succeeds U1 small nuclear ribonucleoprotein particle at 5′ splice sites. Mol. Cell. Biol. *18*, 7510–7520.

Owen, N., Zhou, H., Malygin, A.A., Sangha, J., Smith, L.D., Muntoni, F., and Eperon, I.C. (2011). Design principles for bifunctional targeted oligonucleotide enhancers of splicing. Nucleic Acids Res. *39*, 7194–7208.

Pan, Q., Shai, O., Lee, L.J., Frey, B.J., and Blencowe, B.J. (2008). Deep surveying of alternative splicing complexity in the human transcriptome by high-throughput sequencing. Nat. Genet. *40*, 1413–1415.

Pandit, S., Zhou, Y., Shiue, L., Coutinho-Mansfield, G., Li, H., Qiu, J., Huang, J., Yeo, G.W., Ares, M., Jr., and Fu, X.D. (2013). Genome-wide analysis reveals SR protein cooperation and competition in regulated splicing. Mol. Cell *50*, 223–235.

Pedrotti, S., Bielli, P., Paronetto, M.P., Ciccosanti, F., Fimia, G.M., Stamm, S., Manley, J.L., and Sette, C. (2010). The splicing regulator Sam68 binds to a novel exonic splicing silencer and functions in SMN2 alternative splicing in spinal muscular atrophy. EMBO J. *29*, 1235–1247.

Perrett, A.J., Dickinson, R.L., Krpetić, Z., Brust, M., Lewis, H., Eperon, I.C., and Burley, G.A. (2013). Conjugation of PEG and gold nanoparticles to increase the accessibility and valency of tethered RNA splicing enhancers. Chem. Sci. *4*, 257–265.

Ray, D., Kazan, H., Cook, K.B., Weirauch, M.T., Najafabadi, H.S., Li, X., Gueroussov, S., Albu, M., Zheng, H., Yang, A., et al. (2013). A compendium of RNA-binding motifs for decoding gene regulation. Nature *499*, 172–177.

Rigo, F., Hua, Y., Krainer, A.R., and Bennett, C.F. (2012). Antisense-based therapy for the treatment of spinal muscular atrophy. J. Cell Biol. *199*, 21–25.

Robberson, B.L., Cote, G.J., and Berget, S.M. (1990). Exon definition may facilitate splice site selection in RNAs with multiple exons. Mol. Cell. Biol. *10*, 84–94.

Roca, X., Krainer, A.R., and Eperon, I.C. (2013). Pick one, but be quick: 5′ splice sites and the problems of too many choices. Genes Dev. *27*, 129–144.

Ruskin, B., Zamore, P.D., and Green, M.R. (1988). A factor, U2AF, is required for U2 snRNP binding and splicing complex assembly. Cell *52*, 207–219.

Samatanga, B., Dominguez, C., Jelesarov, I., and Allain, F.H. (2013). The high kinetic stability of a G-quadruplex limits hnRNP F qRRM3 binding to G-tract RNA. Nucleic Acids Res. *41*, 2505–2516.

Sanford, J.R., Wang, X., Mort, M., Vanduyn, N., Cooper, D.N., Mooney, S.D., Edenberg, H.J., and Liu, Y. (2009). Splicing factor SFRS1 recognizes a functionally diverse landscape of RNA transcripts. Genome Res. *19*, 381–394.

Shen, H., and Green, M.R. (2006). RS domains contact splicing signals and promote splicing by a common mechanism in yeast through humans. Genes Dev. *20*, 1755–1765.

Silva, J.C., Gorenstein, M.V., Li, G.Z., Vissers, J.P., and Geromanos, S.J. (2006). Absolute quantification of proteins by LCMSE: a virtue of parallel MS acquisition. Mol. Cell. Proteomics *5*, 144–156.

Singh, R.K., and Cooper, T.A. (2012). Pre-mRNA splicing in disease and therapeutics. Trends Mol. Med. *18*, 472–482.

Singh, N.K., Singh, N.N., Androphy, E.J., and Singh, R.N. (2006). Splicing of a critical exon of human Survival Motor Neuron is regulated by a unique silencer element located in the last intron. Mol. Cell. Biol. *26*, 1333–1346.

Skordis, L.A., Dunckley, M.G., Yue, B., Eperon, I.C., and Muntoni, F. (2003). Bifunctional antisense oligonucleotides provide a trans-acting splicing enhancer that stimulates SMN2 gene expression in patient fibroblasts. Proc. Natl. Acad. Sci. USA *100*, 4114–4119.

Smith, P.J., Zhang, C., Wang, J., Chew, S.L., Zhang, M.Q., and Krainer, A.R. (2006). An increased specificity score matrix for the prediction of SF2/ASF-specific exonic splicing enhancers. Hum. Mol. Genet. *15*, 2490–2508.

Staknis, D., and Reed, R. (1994). SR proteins promote the first specific recognition of Pre-mRNA and are present together with the U1 small nuclear

ribonucleoprotein particle in a general splicing enhancer complex. Mol. Cell. Biol. *14*, 7670–7682.

Tacke, R., and Manley, J.L. (1995). The human splicing factors ASF/SF2 and SC35 possess distinct, functionally significant RNA binding specificities. EMBO J. *14*, 3540–3551.

Uesugi, S., Liu, H., Kugimiya, A., Matsugami, A., and Katahira, M. (2003). RNA and DNA, which contain two GGAGG segments connected with UUUU or TTTT sequences, form entirely different quadruplex structures. Nucleic Acids Res. Suppl., 51–52.

Valcárcel, J., Gaur, R.K., Singh, R., and Green, M.R. (1996). Interaction of U2AF65 RS region with pre-mRNA branch point and promotion of base pairing with U2 snRNA [corrected]. Science *273*, 1706–1709.

Vaughn, J.P., Creacy, S.D., Routh, E.D., Joyner-Butt, C., Jenkins, G.S., Pauli, S., Nagamine, Y., and Akman, S.A. (2005). The DEXH protein product of the DHX36 gene is the major source of tetramolecular quadruplex G4-DNA resolving activity in HeLa cell lysates. J. Biol. Chem. *280*, 38117–38120.

Vezain, M., Saugier-Veber, P., Goina, E., Touraine, R., Manel, V., Toutain, A., Fehrenbach, S., Frébourg, T., Pagani, F., Tosi, M., and Martins, A. (2010). A rare SMN2 variant in a previously unrecognized composite splicing regulatory element induces exon 7 inclusion and reduces the clinical severity of spinal muscular atrophy. Hum. Mutat. *31*, E1110–E1125.

Wang, Z., Hoffmann, H.M., and Grabowski, P.J. (1995). Intrinsic U2AF binding is modulated by exon enhancer signals in parallel with changes in splicing activity. RNA *1*, 21–35.

Wang, E.T., Sandberg, R., Luo, S., Khrebtukova, I., Zhang, L., Mayr, C., Kingsmore, S.F., Schroth, G.P., and Burge, C.B. (2008). Alternative isoform regulation in human tissue transcriptomes. Nature *456*, 470–476.

Wu, J.Y., and Maniatis, T. (1993). Specific interactions between proteins implicated in splice site selection and regulated alternative splicing. Cell *75*, 1061–1070.

Xiao, X., Wang, Z., Jang, M., Nutiu, R., Wang, E.T., and Burge, C.B. (2009). Splice site strength-dependent activity and genetic buffering by poly-G runs. Nat. Struct. Mol. Biol. *16*, 1094–1100.

Zamore, P.D., and Green, M.R. (1989). Identification, purification, and biochemical characterization of U2 small nuclear ribonucleoprotein auxiliary factor. Proc. Natl. Acad. Sci. USA *86*, 9243–9247.

Zhu, J., and Krainer, A.R. (2000). Pre-mRNA splicing in the absence of an SR protein RS domain. Genes Dev. *14*, 3166–3178.

Cell Reports, Volume *9*

Supplemental Information

A Targeted Oligonucleotide Enhancer of SMN2 Exon 7 Splicing Forms Competing Quadruplex and Protein Complexes in Functional Conditions

Lindsay D. Smith, Rachel L. Dickinson, Christian M. Lucas, Alex Cousins, Alexey A. Malygin, Carika Weldon, Andrew J. Perrett, Andrew R. Bottrill, Mark S. Searle, Glenn A. Burley, and Ian C. Eperon

Supplemental Figure 1

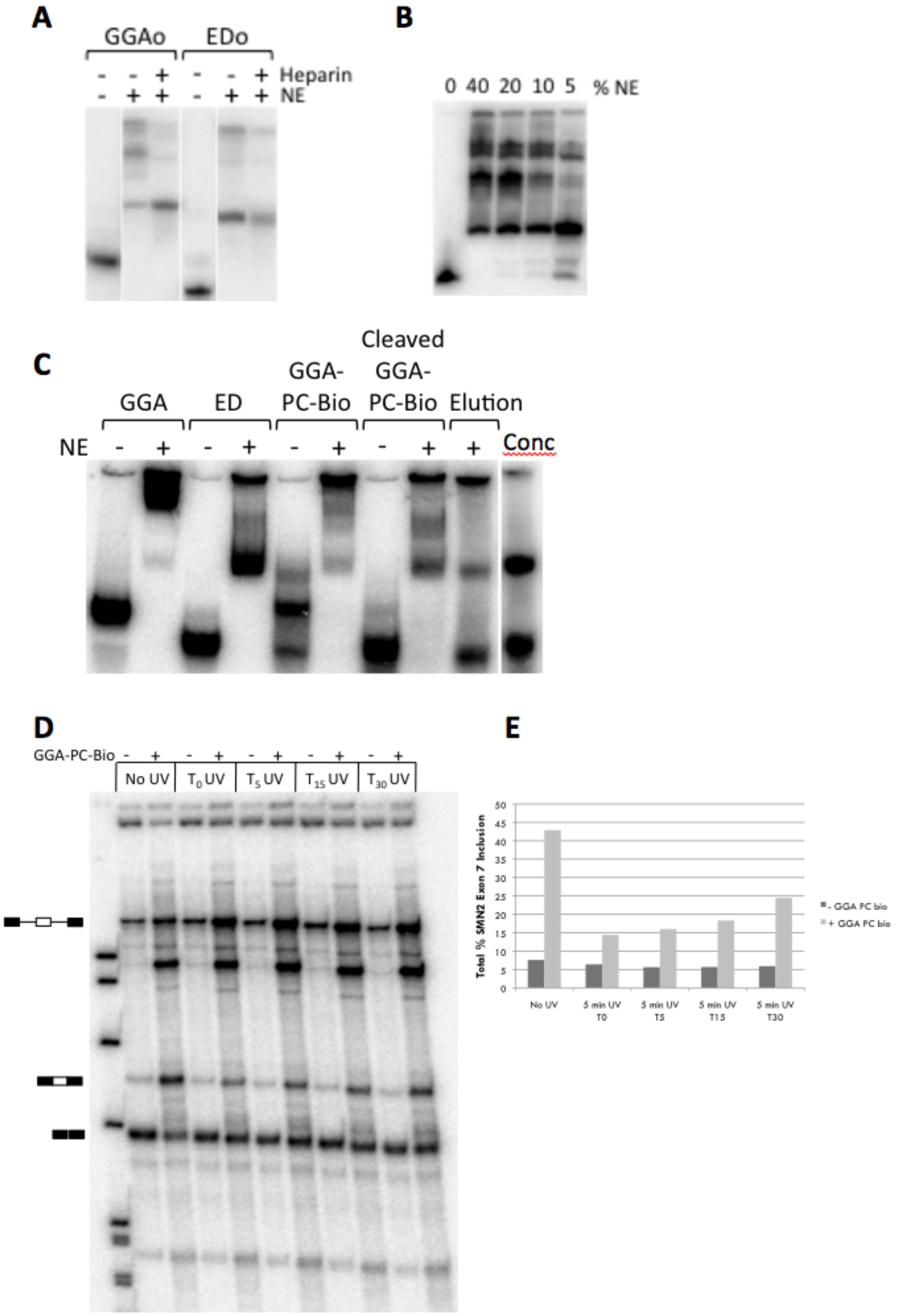

Supplemental Figure 1. Complex formation on photocleavable oligonucleotide. Related to Figure 4.

(A) Formation of heparin-resistant complexes on the ESE domain. Native gel electrophoresis of complexes formed by labelled 2'-O-methyl derivatives of GGA and ED after incubation in nuclear extract (NE) and then heparin, as indicated.

(B) Native gel electrophoresis of complexes formed in various concentrations (%v/v) of nuclear extract.

(C) The ESE domain of GGA forms a complex in nuclear extract equivalent to that formed by ED. A GGA derivative containing a photocleavable linkage between AD and ED (GGA-PC-Bio) was incubated in nuclear extract and irradiated with long wave UV light (for cleavage) prior to native gel electrophoresis. The sample in the right-hand lane was incubated with Neutravidin beads and washed prior to cleavage and analysis of the eluted complex. Conc, eluted material after concentration by ultrafiltration.

(D) Effects of photocleavage of GGA-PC-Bio at different times after initiation of splicing reactions with SMN2 exon 7-containing pre-mRNA. Splicing reactions were incubated for 2 h. Irradiation was done at the times indicated from the start of the reaction.

(E) Level of exon inclusion (inclusion mRNA isoform x 100/[inclusion + exclusion isoforms]) in (D), showing that irradiation did not affect the low level of inclusion in the absence of a TOES oligonucleotide and that its effects on reactions in the presence of GGA-PC-Bio were greater with earlier times of irradiation.

Supplemental Figure 2

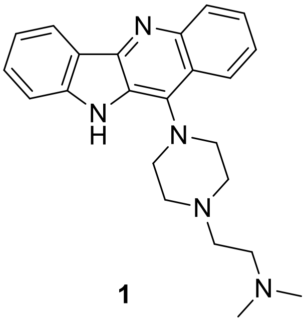

1

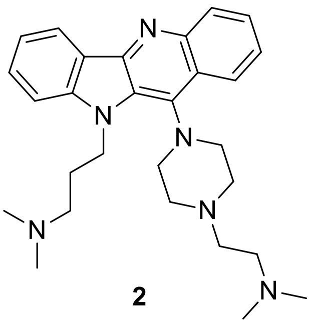

2

Supplemental Figure 2. Structures of quadruplex stabilizers. Related to Figure 6A,B. (1), GSA-0820; (2), GSA-0902. (Boddupally et al., 2012)

Supplemental Figure 3

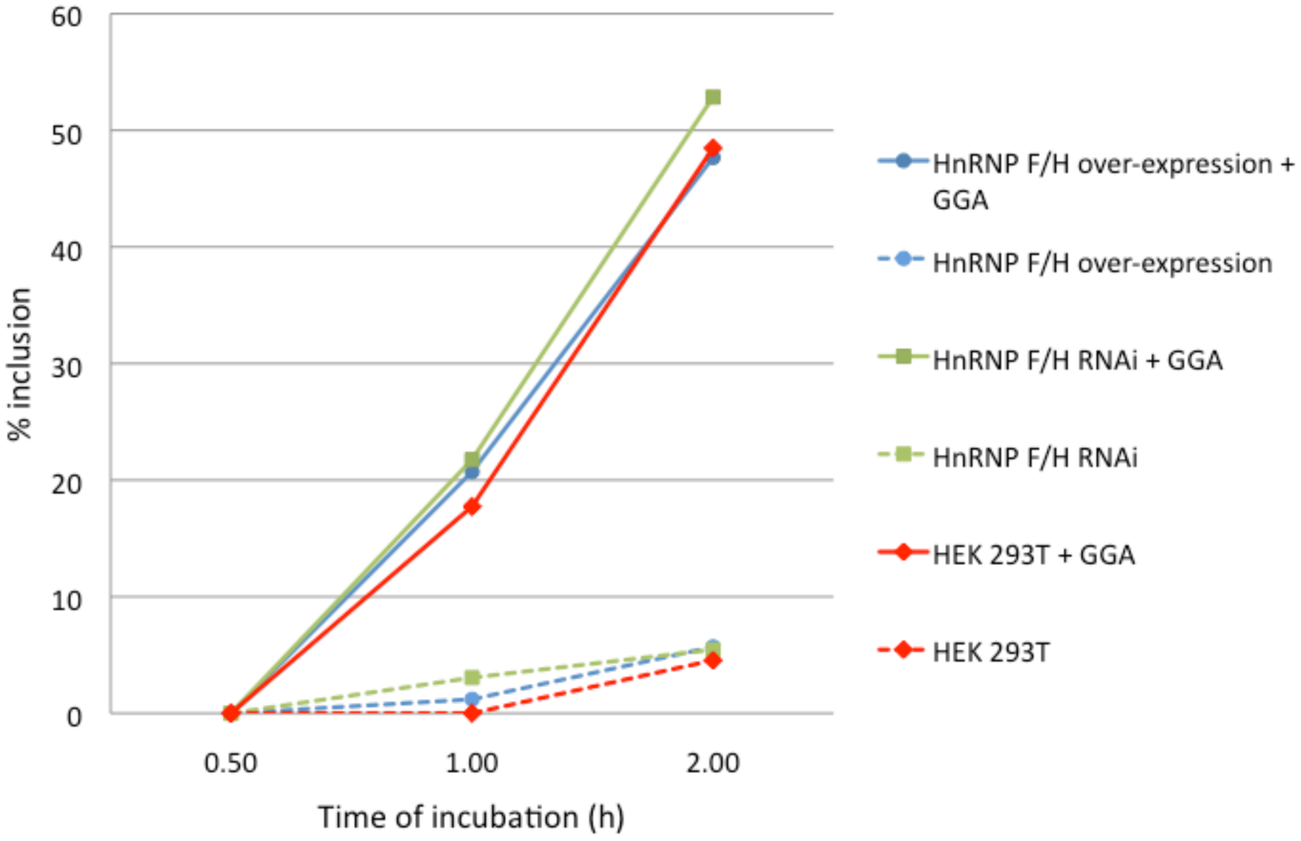

Supplemental Figure 3. Effects of hnRNP F/H depletion and over-expression on activity of the GGA oligonucleotide. Related to Figure 6C. The percentage of inclusion of SMN2 exon 7 was calculated from the phosphor image shown in Figure 6C, where % inclusion is [100*inclusion/(inclusion + exclusion)]. The value changes with time of incubation, probably because the formation of inclusion mRNA (requiring two splicing reactions) is slower than the formation of the skipped or excluded isoform.

Supplemental Table 1.

Rank	Identified Proteins	Accession Number	Mr kDa	Cover-age(%)
1	Probable ATP-dependent RNA helicase DHX36	Q9H2U1	115	53
2	5'-3' exoribonuclease 2	Q9H0D6	109	53
3	Nucleolin	P19338	77	33
4	HnRNP H	G8JLB6 (+1)	51	51
5	HnRNP U-like protein 1	B7Z4B8 (+3)	86	40
6	HnRNP U	Q00839	91	40
7	HnRNP A1	F8VRQ1 (+4)	33	55
8	Double-stranded RNA-binding protein 76	C9JFV5 (+7)	83	40
9	Serine/arginine-rich splicing factor 1	Q07955	28	46
10	Isoform 4 of Cellular nucleic acid-binding protein	P62633-4	20	60
11	CDKN2A-interacting protein	Q9NXV6	61	58
12	HnRNPs A2/B1	P22626 (+1)	37	51
13	Procollagen-lysine,2-oxoglutarate 5-dioxygenase 1	Q02809	84	41
14	HnRNP A3	E7ERJ4 (+2)	34	37
15	RNA-binding protein 45	Q8IUH3 (+1)	54	37
16	Interleukin enhancer-binding factor 2	Q12905	43	28
17	HnRNP H2	P55795	49	47
18	HCG2044799	H3BQZ7 (+1)	85	19
19	Transcriptional activator protein Pur-alpha	Q00577	35	32
20	RNA-binding protein FUS	B4DR70 (+2)	45	27
21	HnRNP F	P52597	46	30
22	5'-3' exoribonuclease 1	Q8IZH2 (+1)	194	6.9
23	Transcriptional activator protein Pur-beta	Q96QR8	33	28
24	U1 small nuclear ribonucleoprotein A	P09012	31	13
25	Serine/arginine-rich splicing factor 2	Q01130 (+1)	25	22
26	TAR DNA-binding protein 43	Q13148 (+1)	45	15
27	HnRNP Q	O60506 (+3)	70	12
28	Serine/arginine-rich splicing factor 5	Q13243	31	20
29	Nuclease-sensitive element-binding protein 1	P67809	36	32
30	Putative ATP-dependent RNA helicase DHX15	F5H6K0 (+1)	90	5.9
31	HnRNP D-like	O14979 (+2)	46	5.2
32	Serine/arginine-rich splicing factor 9	Q13242	26	22
33	Serine/arginine-rich-splicing factor 7	C9JAB2 (+4)	27	13
34	HnRNP R	O43390	71	10
35	Probable ATP-dependent RNA helicase DDX5	E7ETL9 (+1)	62	4.6
36	Serine/arginine-rich splicing factor 6	Q13247 (+1)	40	7.3
37	General transcription factor II-I	B4DH52 (+4)	112	2.2
38	Small nuclear ribonucleoprotein Sm D3	B4DJP7 (+1)	13	16
39	Splicing factor 3B subunit 2	E9PJ04 (+4)	39	9.3
40	TATA-binding protein-associated factor 2N	Q92804 (+1)	62	11
41	Thyroid hormone receptor-associated protein 3	Q9Y2W1	109	2.2

Supplemental Table 1. Ranking of proteins associated with the ED of the GGA oligonucleotide after its release from the AD by photocleavage. Related to Figure 4. Ranking

was done using Top3TIC on Scaffold Viewer, after removal of proteins also identified in a control (-oligonucleotide) preparation. The coverage results refer to the proportion of each protein represented in identified peptides. The probability of identification of each protein was >99.9%.

Supplemental Table 2

	**-**	**H RNAi**	**F/H RNAi**	**GFP-H**
High Mr	28	39	36	19
GFP-H				26
hnRNP F/H	41	24	22	33
SRSF1	19	23	28	10
CNBP	12	13	15	12

Supplemental Table 2. Relative intensities of proteins crosslinked to 5'-end labelled ED domain in functional HEK293T nuclear extracts. Related to Figure 4D. The values represent the percentage in each band of the total radioactivity in the identified bands.

Supplemental Table 3

**ESE sequence**	**T_m (fold) (^oC)**	**T_m (melt) (^oC)**
23-mer ESE (2'Ome)	57.5 ±0.1	60.6 ±0.1
23-mer ESE (2'OMe/PS)	66.3 ±0.3	64.9 ±0.2
23-mer ESE (unmodified RNA)	62.2 ±0.1	76.0 ±0.4
15-mer ESE (unmodified RNA)	63.0 ±0.1	75.0 ±0.1

Supplemental Table 3. Apparent T_m values for ESE analogues from CD melting and refolding curves. Related to Figure 5C. Values were recorded at the wavelengths shown in Figure 5C. Transition midpoints were determined based on an apparent two-state model.

Supplemental Experimental Procedures

Protein digestion, analysis by mass spectrometry and data processing. Proteomics was carried out by the University of Leicester Proteomics Facility (PNACL, University of Leicester). Bands of interest were excised from the gel, and in-gel trypsin digestion was carried out upon each (Speicher et al., 2000). Each slice was destained using 200mM ammonium bicarbonate/20% acetonitrile, followed by reduction (10 mM dithiothreitol, Melford Laboratories Ltd., Suffolk, UK), alkylation (100 mM iodoacetamide, Sigma, Dorset, UK) and enzymatic digestion with trypsin (sequencing grade modified porcine trypsin, Promega, Southampton, UK) in 50mM triethylammonium bicarbonate (Sigma) using an automated digest robot (Multiprobe II Plus EX, Perkin Elmer, UK). After overnight digestion, samples were acidified using formic acid (final concentration 0.1%).

LC-MS/MS was carried out using an RSLCnano HPLC system (Dionex, UK) and an LTQ-Orbitrap-Velos mass spectrometer (Thermo Scientific). Samples were loaded at high flow rate onto a reverse-phase trap column (0.3mm i.d. x 1mm), containing 5μm C18 300 Å Acclaim PepMap media (Dionex) maintained at a temperature of 37 °C. The loading buffer was 0.1% formic acid / 0.05% trifluoroacetic acid / 2% acetonitrile. Peptides were eluted from the trap column at a flow rate of 0.3 μl/min and through a reverse-phase capillary column (75μm i.d. x 250mm) containing Symmetry C18 100 Å media (Waters, UK) that was manufactured in-house using a high pressure packing device (Proxeon Biosystems, Denmark). The output from the column was sprayed directly into the nanospray ion source of the LTQ-Orbitrap-Velos mass spectrometer.

The LTQ-Orbitrap-Velos mass spectrometer was set to acquire a 1 microscan FTMS scan event at 60000 resolution over the m/z range 350-1250 Da in positive ion mode. Accurate calibration of the FTMS scan was achieved using a background ion lock mass for polydimethylcyclosiloxane (445.120025 Da). Subsequently up to 10 data dependent HCD MS/MS were triggered from the FTMS scan. The isolation width was 2.0 Da, normalized collision energy 40.0, Activation time 10 ms. Dynamic exclusion was enabled. The .raw data file obtained from each LC-MS/MS acquisition was processed using Proteome Discoverer (version 1.4, Thermo Scientific), searching each file in turn using Mascot (Perkins et al., 1999) (version 2.2.04, Matrix Science Ltd.) against the UniProtKB-Swissprot database. The peptide tolerance was set to 5 ppm and the MS/MS tolerance was set to 0.02 Da. Fixed modifications were set as carbamidomethyl (C) with variable modifications of oxidation (M). A decoy database search was performed. The output from Proteome Discoverer was further processed using Scaffold Q+S (Searle, 2010) (version 3.6.1, Proteome Software). Upon import, the data was searched using X!Tandem (Craig and Beavis, 2004) (The Global Proteome Machine Organization). PeptideProphet (Keller et al., 2002) and ProteinProphet (Nesvizhskii et al., 2003) (Institute for Systems Biology) probability thresholds of

95% were calculated from the decoy searches and Scaffold was used to calculate an improved 95% peptide and protein probability threshold based on the data from the two different search algorithms. Candidate proteins were only accepted if they contained at least 3 peptides >95%. Scaffold was used to calculate the Total Ion Count for the 3 most abundant peptides of each protein for the purposes of protein quantitation.

Structural analysis of oligonucleotides. Unmodified 15-mer and 23-mer samples were from Ribotask (Denmark) and Dharmacon (USA). 2'OMe RNA oligonucleotides were synthesized from DMT-protected β-(cyanoethyl) phosphoramidites (Link Technologies, USA) on 1 μmol CPG supports using an Applied Biosystems 394 nucleic acid synthesizer. RNA oligonucleotides were synthesized from TC-protected phosphoramidites (Link Technologies) (Dellinger et al., 2011). After synthesis, 2'OMe sequences were released by aminolysis (35% aqueous ammonia, 4 h at ambient temperature) and deprotected by heating in 35% aqueous ammonia solution at 55°C for 16h. RNA oligonucleotides were treated in diethylamine/acetonitrile and deprotected with ethylene diamine/toluene prior to elution with triethylammonium acetate. All oligonucleotides were purified by electrophoresis on 10% denaturing gels. Phosphorothioate oligonucleotides were prepared as described (Krotz et al., 2004; Ravikumar et al., 2006). All products were desalted using Illustra NAP™ 25 columns, lyophilised and dissolved in double-distilled H_2O.

For structural analysis, samples were reconstituted in KCl buffer (100mM KCl, 10mM K_2HPO_4/KH_2PO_4, pH 7) made with filtered and degassed water. Prior to use samples were heated to 95°C for 10 minutes and then kept at room temperature for at least an hour to allow secondary structure to form. Unmodified RNA sample concentration was estimated using the nearest neighbour method. CD, UV and thermal denaturation spectra were recorded using a PiStar 180 Spectrophotometer (Applied Photophysics, UK) with strand concentrations between 1-10 μM. Temperature ramping used a rate of 3 min/0.5^{o}C steps and a tolerance of 0.2^{o} C. Data was fitted in OriginPro 8.6 (OriginLab). For NMR, samples were diluted with 10% D_2O to 0.3 to 0.8 mM. Spectra were recorded using a Bruker Avance 600MHz and Bruker Avance III 800MHz with Cryoprobe. The pulse program used was a 1D sequence with water suppression and excitation sculpting with gradients (Hwang and Shaka, 1995). Data was processed in Bruker Topspin 3.1. Mass spectrometry was done in negative ion mode using a Synapt High Definition Mass Spectrometer with Quadrupole TOF mass analyzer (Waters, UK). Samples used in mass spectrometry were buffer-exchanged into 150 mM NH_4OAc, either using a polyacrylamide desalting column (Thermo Scientific) followed by spin concentration to 20-40 μM (23-mer), or dialysis (Slide-a-Lyzer G2, Thermo Scientific, 2K MWCO) to 31 μM (15-mer). Samples were mixed with 10% MeOH immediately before analysis. An injection rate of 4-10 uL.min^{-1} was used and spectra recorded with capillary voltage 1.5-2.5 kV. Data was processed with MassLynx V4.1 (Waters) with smoothing and baseline subtraction.

References

Boddupally, P.V., Hahn, S., Beman, C., De, B., Brooks, T.A., Gokhale, V., and Hurley, L.H. (2012). Anticancer activity and cellular repression of c-MYC by the G-quadruplex-stabilizing 11-piperazinylquindoline is not dependent on direct targeting of the G-quadruplex in the c-MYC promoter. J Med Chem *55*, 6076-6086.

Craig, R., and Beavis, R.C. (2004). TANDEM: matching proteins with tandem mass spectra. Bioinformatics *20*, 1466-1467.

Dellinger, D.J., Timar, Z., Myerson, J., Sierzchala, A.B., Turner, J., Ferreira, F., Kupihar, Z., Dellinger, G., Hill, K.W., Powell, J.A., *et al.* (2011). Streamlined process for the chemical synthesis of RNA using 2'-O-thionocarbamate-protected nucleoside phosphoramidites in the solid phase. J. Am. Chem. Soc. *133*, 11540-11556.

Hwang, T.L., and Shaka, A.J. (1995). Water Suppression That Works - Excitation Sculpting Using Arbitrary Wave-Forms and Pulsed-Field Gradients. Journal of Magnetic Resonance Series A *112*, 275-279.

Keller, A., Nesvizhskii, A.I., Kolker, E., and Aebersold, R. (2002). Empirical statistical model to estimate the accuracy of peptide identifications made by MS/MS and database search. Anal Chem *74*, 5383-5392.

Krotz, A.H., Gorman, D., Mataruse, P., Foster, C., Godbout, J.D., Coffin, C.C., and Scozzari, A.N. (2004). Phosphorothioate oligonucleotides with low phosphate diester content: Greater than 99.9% sulfurization efficiency with "aged" solutions of phenylacetyl disulfide (PADS). Org. Proc. Res. Dev. *8*, 852-858.

Nesvizhskii, A.I., Keller, A., Kolker, E., and Aebersold, R. (2003). A statistical model for identifying proteins by tandem mass spectrometry. Anal Chem *75*, 4646-4658.

Perkins, D.N., Pappin, D.J., Creasy, D.M., and Cottrell, J.S. (1999). Probability-based protein identification by searching sequence databases using mass spectrometry data. Electrophoresis *20*, 3551-3567.

Ravikumar, V.T., Andrade, M., Carty, R.L., Dan, A., and Barone, S. (2006). Development of siRNA for therapeutics: efficient synthesis of phosphorothioate RNA utilizing phenylacetyl disulfide (PADS). Bioorg. Med. Chem. Lett. *16*, 2513-2517.

Searle, B.C. (2010). Scaffold: a bioinformatic tool for validating MS/MS-based proteomic studies. Proteomics *10*, 1265-1269.

Speicher, K.D., Kolbas, O., Harper, S., and Speicher, D.W. (2000). Systematic analysis of peptide recoveries from in-gel digestions for protein identifications in proteome studies. J Biomol Tech *11*, 74-86.
